# Cortical branched actin determines cell cycle progression

**DOI:** 10.1038/s41422-019-0160-9

**Published:** 2019-04-10

**Authors:** Nicolas Molinie, Svetlana N. Rubtsova, Artem Fokin, Sai P. Visweshwaran, Nathalie Rocques, Anna Polesskaya, Anne Schnitzler, Sophie Vacher, Evgeny V. Denisov, Lubov A. Tashireva, Vladimir M. Perelmuter, Nadezhda V. Cherdyntseva, Ivan Bièche, Alexis M. Gautreau

**Affiliations:** 1 BIOC, Ecole polytechnique, CNRS, IP Paris, Palaiseau, France; 2 N.N. Blokhin National Medical Research Center of Oncology, Moscow, Russia; 3 Department of Genetics, Institut Curie, Paris, France; 4 Tomsk National Research Medical Center, Tomsk, Russia; 5 Tomsk State University, Tomsk, Russia; 6 School of Biological and Medical Physics, Moscow Institute of Physics and Technology, Dolgoprudny, Russia

**Keywords:** Actin, Lamellipodia, Breast cancer

## Abstract

The actin cytoskeleton generates and senses forces. Here we report that branched actin networks from the cell cortex depend on ARPC1B-containing Arp2/3 complexes and that they are specifically monitored by type I coronins to control cell cycle progression in mammary epithelial cells. Cortical ARPC1B-dependent branched actin networks are regulated by the RAC1/WAVE/ARPIN pathway and drive lamellipodial protrusions. Accordingly, we uncover that the duration of the G1 phase scales with migration persistence in single migrating cells. Moreover, cortical branched actin more generally determines S-phase entry by integrating soluble stimuli such as growth factors and mechanotransduction signals, ensuing from substratum rigidity or stretching of epithelial monolayers. Many tumour cells lose this dependence for cortical branched actin. But the RAC1-transformed tumour cells stop cycling upon Arp2/3 inhibition. Among all genes encoding Arp2/3 subunits, *ARPC1B* overexpression in tumours is associated with the poorest metastasis-free survival in breast cancer patients. Arp2/3 specificity may thus provide diagnostic and therapeutic opportunities in cancer.

## Introduction

Actin polymerises either in a linear or in a branched manner. Whereas formins polymerise linear actin, the Arp2/3 complex polymerises branched actin in the cell. Branched actin networks develop a pushing force, which promotes protrusion of the plasma membrane or fission of intracellular membranes.^1^ The Arp2/3 complex is activated by Nucleation Promoting Factors (NPFs) and a pre-existing actin filament, onto which the Arp2/3 complex must land to induce the growth of a “daughter filament” from the side of the “mother filament”.^2^ The Arp2/3 complex itself thus creates the branching junction of two actin filaments. The Arp2/3 complex is activated at different subcellular locations by various NPFs.^1^ For example, the WAVE complex is critical for the formation of lamellipodia, whereas the WASH complex is critical for the scission of transport intermediates from endosomes.

During cell migration, the leading edge protrudes as an adherent structure called a lamellipodium. At the lamellipodium edge, Arp2/3 complexes are activated by WAVE complexes, whose recruitment and activation depend on the small GTPase RAC1.^3^ RAC1 signalling also activates the Arp2/3 inhibitory protein ARPIN, which terminates the protrusion.^4^ This antagonistic Arp2/3 regulation by RAC1 thus regulates the protrusion lifetime. With a long lifetime, the protrusion develops into a lamellipodium that mediates persistent migration;^5^ with a short lifetime, branched actin contributes to the actin cortex and to limited membrane fluctuations.^6,7^

When investigating what determines the lifetime of protrusions, we revisited well-known inducers of lamellipodia. Starved cells are immobile and arrested in the cell cycle. When stimulated by growth factors, they develop lamellipodia, and resume cell cycle progression (Supplementary information, Fig. S1a). Confluent cells are also immobile and arrested in the cell cycle. A wound in the monolayer induces cells of the periphery to develop lamellipodia and to resume cell cycle progression (Supplementary information, Fig. S1b). Both migration and proliferation contribute to wound healing. These examples illustrate a possible coupling between migration and cell cycle progression and raise the question of whether actin polymerisation is involved in the regulation of cell cycle progression.

## Results

### Cortical branched actin controls cell cycle progression

**Fig. 1:**
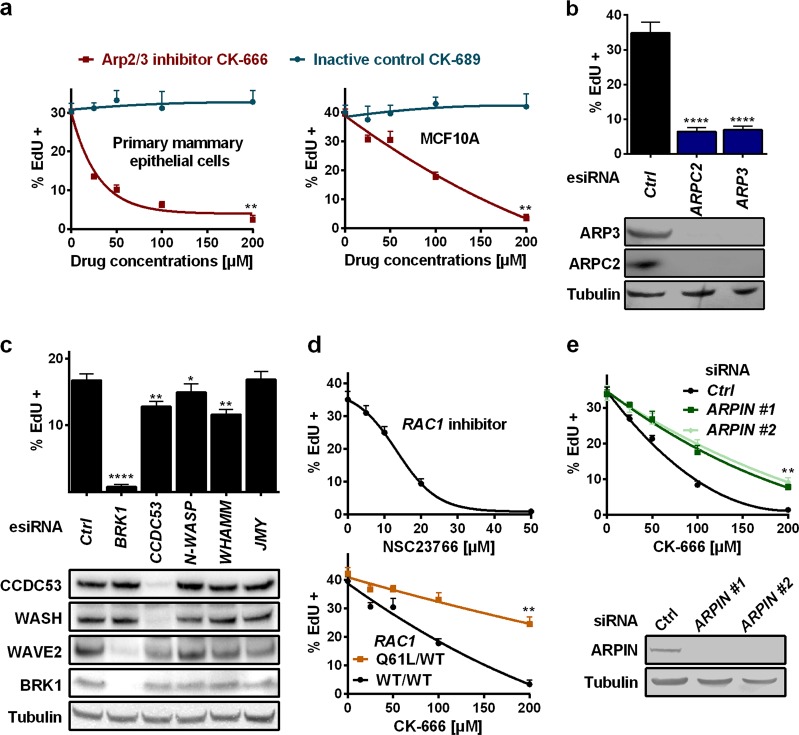
Delineation of the actin structure that controls cell cycle progression. **a** The CK-666 drug that blocks Arp2/3 activation, but not the inactive control, CK-689, prevents cell cycle progression of primary mammary epithelial cells and of mammary immortalised MCF10A cells, which are then used throughout the work except when otherwise indicated. Drug treatments are for 16 h. The number of cells in S phase is monitored by the incorporation of the thymidine analogue, EdU, provided to the cells for 1 h. **b** Depletion of the Arp2/3 complex, through two of its subunits, ARPC2 and ARP3, blocks S-phase entry. **c** Depletion of all known NPFs, the Arp2/3 activators. The WAVE complex targeted through its subunit BRK1, but not the WASH complex targeted through its subunit CCDC53, nor the others, is critical for cell cycle progression. **d** The drug NSC23766 that impairs RAC1 activation impedes S-phase entry in a dose-dependent manner; genome-edited MCF10A cells where one *RAC1* allele encodes the constitutively active Q61L form are less sensitive to Arp2/3 inhibition than WT cells. **e** Cells depleted of the Arp2/3 inhibitory protein, ARPIN, are also less sensitive to Arp2/3 inhibition than control cells. Data are mean ± s.e.m of five technical repeats; one experiment representative of two biological repeats is displayed; Mann–Whitney test at 200 µM of drug for **a**, **d** and **e**, one-way ANOVA followed by Dunnett’s post-Hoc test for **b** and **c**

Using the human MCF10A breast immortalised cell line and EdU incorporation during S phase as a read-out for cell cycle progression, we found that two drugs, Cytochalasin D and Latrunculin A, that block actin polymerisation, stopped the cell cycle in a dose-dependent manner (Supplementary information, Fig. S2), as reported decades ago in mouse embryonic fibroblasts.^8^ Strikingly, the cell cycle block was also obtained with two structurally unrelated Arp2/3 inhibitors,^9^ but not with the formin inhibitor SMIFH2 (Fig. 1a; Supplementary information, Fig. S2). Arp2/3 inhibition arrested cells in G1 (Supplementary Information, Fig. S3). The G1 block was reversed upon drug washout indicating that the block of cell proliferation was not due to apoptosis, nor senescence. Arp2/3 inhibition did not impair cytokinesis during mitosis of MCF10A cells (Supplementary Information, Fig. S3e, Movie S1). Primary mammary epithelial cells also blocked cell cycle progression in response to Arp2/3 inhibition (Fig. 1a), indicating that this effect was not associated with the immortalisation of the MCF10A cell line. Furthermore, RNAi-mediated depletion of the Arp2/3 complex, using esiRNAs (see Material and Methods), blocked cell cycle progression, just like drug-mediated inhibition (Fig. 1b). All together, these results strongly suggest that branched actin structures generated by the Arp2/3 complex are required for the G1-S transition of the cell cycle.

To look for a possible specificity in the branched actin structures playing this role, we inactivated each known NPF by targeting the BRK1 subunit of the WAVE complex, the CCDC53 subunit of the WASH complex, the ubiquitous N-WASP and the two paralogous WHAMM and JMY proteins.^1^ Only WAVE complex depletion led to a severe cell cycle arrest, similar to Arp2/3 depletion (Fig. 1c; Supplementary information, Figs. S1c, S4). Since the activity of WAVE depends on the RAC1 GTPase, the master inducer of lamellipodia, we inhibited RAC1 using the NSC23766 compound and this treatment indeed prevented S-phase entry (Fig. 1d). To confirm the role of RAC1, we used a genome-edited MCF10A cell line, where one *RAC1* allele encodes the constitutively active, GTPase-defective, Q61L mutant. Importantly, cell cycle progression of RAC1 Q61L-expressing cells was potentiated in this case, since a higher dose of the Arp2/3 drug was required to achieve the same level of inhibition in these cells as compared to wild type cells. Depletion of ARPIN, which antagonizes WAVE, also potentiated cell cycle progression (Fig. 1e). These results establish that branched actin networks polymerised by the RAC1-WAVE pathway, and antagonised by ARPIN, deliver the essential signal for cell cycle progression. But how does the cell discriminate cortical branched actin networks from the other ones involved in intracellular trafficking?

### Specific sensing of cortical branched actin

**Fig. 2:**
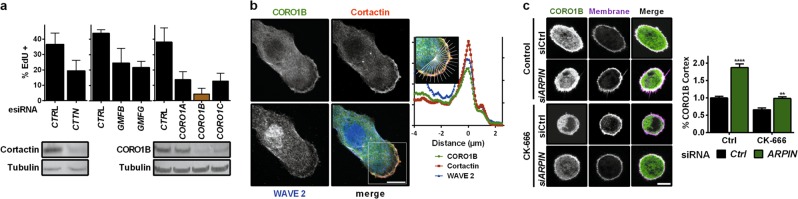
CORO1B specifically senses cortical branched actin. **a** RNAi screen for the proteins that control cell cycle progression among the known proteins that recognise the Arp2/3 complex at the actin branch. *CTTN* encodes Cortactin, *GMFB* and *GMFG* encode the two Glial Maturation Factors β and γ, and *CORO1A*, *CORO1B* and *CORO1C* encode the three type I coronins. Western blot targeting Cortactin and CORO1B validate their depletion. **b** Confocal images of MCF10A expressing GFP-CORO1B stained with antibodies targeting the WAVE2 NPF and the branched actin marker, Cortactin. Sum of radial line scans registered with respect to the cell edge of one lamellipodium (representative of 10 from different cells). **c** Cortical enrichment of CORO1B depends on Arp2/3 activity. Confocal images of MCF10A expressing GFP-CORO1B. Cells were treated with the Arp2/3 inhibitor CK-666 or depleted of ARPIN, as indicated. Data are mean ± s.e.m of five technical repeats (**a**, **c**); two biological repeats with similar results; two-way ANOVA followed by Sidak’s test for **c**. Scale bar: 10 µm

To identify the protein sensing cortical branched actin, we screened all molecules reported to recognise the Arp2/3 complex in the context of the actin branch, namely cortactin, GMFs and type I coronins, which all regulate the stability of the branched junction^10,11^ (Fig. 2a). Only CORO1B was strictly required for cells to cycle. CORO1B decorates lamellipodia (Fig. 2b), as previously reported,^12^ but was absent from the endosomal domain that polymerises branched actin networks,^13–15^ whereas both actin structures were stained by Cortactin (Supplementary information, Fig. S5). CORO1B enrichment at the cortex depended on Arp2/3 activity, being decreased by Arp2/3 inhibition and increased by ARPIN depletion (Fig. 2c). CORO1B thus fulfils the required properties for a specific sensor of cortical branched actin. The next question was what specific cue CORO1B might be sensing at the cortex.

**Fig. 3:**
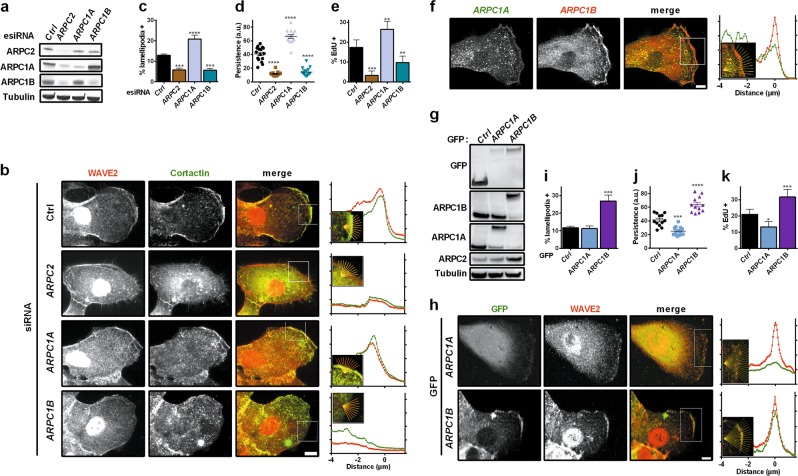
ARPC1B-containing Arp2/3 complexes nucleate the cortical branched actin that drives migration persistence and promotes cell cycle progression. **a** The single Arp2/3 subunit ARPC2 or the two paralogous subunits, ARPC1A and ARPC1B, were depleted using siRNA. The partial down-regulation of ARPC2, upon ARPC1A or ARPC1B depletion, suggests that both ARPC1A- and ARPC1B-containing complexes co-exist in MCF10A cells. **b** Depletion of Arp2/3 complexes containing ARPC1B, but not ARPC1A, impairs the formation of cortical branched actin networks, as indicated by Cortactin staining. **c** Depletion of the Arp2/3 complexes that contain ARPC1B decreases the number of cells displaying lamellipodia, whereas the depletion of complexes containing ARPC1A increases it (*n* > 130 cells). **d** Depletion of ARPC1B decreases migration persistence of MCF10A cells, in line with its effect on lamellipodium formation; depletion of ARPC1A increases migration persistence (*n* *=* 15 cells). **e** ARPC1B depletion decreases cell cycling, whereas ARPC1A depletion increases it. **f** Immunofluorescence of ARPC1A and ARPC1B in MCF10A cells. Endogenous ARPC1B, but not ARPC1A, localises to the lamellipodium. **g** Characterisation of stable MCF10A cell lines expressing GFP-ARPC1A or GFP-ARPC1B by western blot. **h** GFP-ARPC1B, but not GFP-ARPC1A, localises to the lamellipodium. **i** Cells overexpressing GFP-ARPC1B display more lamellipodia compared to controls (*n* > 110 cells). **j** Expression of GFP-ARPC1B increases persistent migration, whereas the expression of GFP-ARPC1A decreases it. **k** ARPC1B expression increases cell cycling, whereas ARPC1A depletion decreases it. Data are mean ± s.e.m; one experiment representative of two biological repeats is displayed, one-way ANOVA followed by Dunnett’s post-Hoc compared to the control for **c**–**e**, **i**–**k**. Confocal microscopy; Scale bar: 5 µm

Type I coronins were reported to partially destabilise actin branches,^10^ depending on the precise composition of paralogous subunits in the Arp2/3 complex.^16^ We thus compared the two ARPC1 paralogous subunits with the single ARPC2 subunit by siRNA-mediated depletion. When ARPC2 was depleted, both ARPC1A and ARPC1B were down-regulated, whereas ARPC2 levels were only modestly affected when either one of the two ARPC1 subunits was depleted (Fig. 3a). This result suggests that ARPC1A- and ARPC1B-containing Arp2/3 complexes co-exist in MCF10A cells. Depletion of ARPC1B, but not of ARPC1A, affected the formation of cortical branched actin networks and lamellipodia, similar to the result of single ARPC2 subunit depletion (Fig. 3b, c; Supplementary information, Fig. S6). In contrast, depletion of ARPC1A, but not of ARPC1B, impaired the formation of branched actin networks at the surface of endosomes (Supplementary information, Fig. S5). These differential requirements of ARPC1A- and ARPC1B-containing complexes were also obvious in migration persistence, which was strongly decreased in ARPC1B-depleted cells (Fig. 3d; Supplementary information, Movie S2). Some level of competition between the two types of Arp2/3 complexes, however, must exist, since migration persistence was increased in ARPC1A-depleted cells. Strikingly, cell cycle progression mirrored migration persistence: S-phase entry was decreased in ARPC1B- and ARPC2-depleted cells and increased in ARPC1A-depleted cells (Fig. 3e). We then examined the cellular localization of ARPC1A and ARPC1B by immunofluorescence using two specific antibodies from different species that allowed simultaneous labelling in the same cells (Supplementary information, Fig. S7). In line with their differential roles, ARPC1B was enriched at the lamellipodium, whereas ARPC1A was mostly labelling dotty internal structures (Fig. 3f).

To confirm these localisations and to complement loss-of-function by gain-of-function experiments, we isolated stable MCF10A lines stably expressing the two GFP-ARPC1 fusion proteins (Fig. 3g). GFP-ARPC1B replaced endogenous ARPC1B and its opposite paralog, ARPC1A. GFP-ARPC1A displayed a similar behavior, albeit less strongly. Indeed, overexpression of ARPC1A is less prominent than that of ARPC1B, as judged by the corresponding increase in ARPC2 levels, which likely reflect total Arp2/3 levels. Consistent with depletion experiments, GFP-ARPC1B was clearly enriched in lamellipodia, whereas GFP-ARPC1A did not display an obvious cortical localisation (Fig. 3h). Moreover, GFP-ARPC1B overexpression increased the number of cells displaying lamellipodia (Fig. 3i). In contrast, at the surface of endosomes, GFP-ARPC1A, but not GFP-ARPC1B, colocalised with the retromer that recruits the WASH complex (Supplementary information, Fig. S5).^15^ Consistently, ARPC1B overexpression strongly increases migration persistence, whereas ARPC1A expression decreases it (Fig. 3j; Supplementary information, Movie S3). EdU incorporation was strongly enhanced by the overexpression of ARPC1B and decreased by the expression of ARPC1A (Fig. 3k). In fact, in all genetic perturbations examined, cell cycle progression appeared coupled to lamellipodium formation and migration persistence (Supplementary information, Fig. S8).

**Fig. 4:**
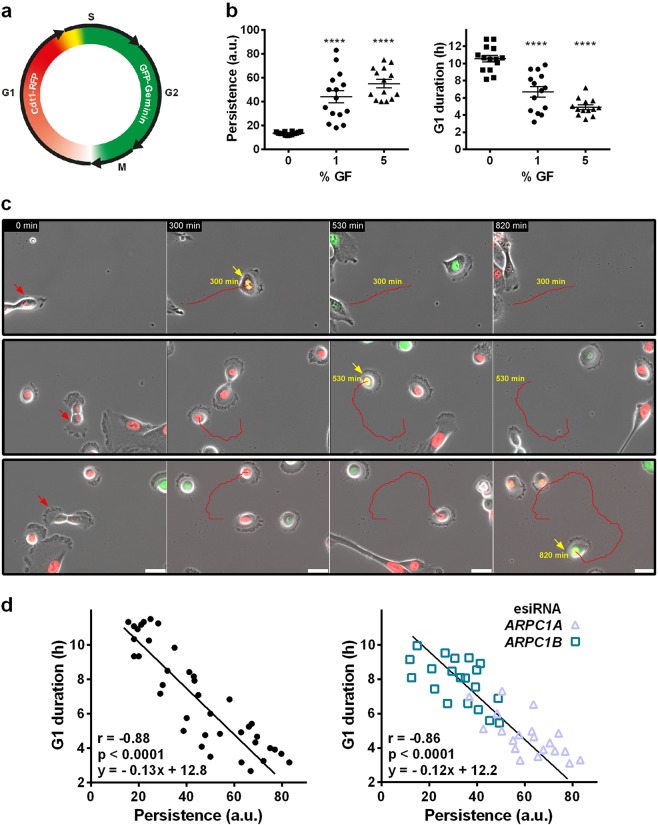
Correlation of G1 phase duration with migration persistence in single cells. **a** A FUCCI reporter cell line was obtained by isolating a stable MCF10A clone expressing GFP-Geminin and Cdt1-RFP. **b** Individual cells were tracked during random migration in either 0, 1 or 5% growth factors (GFs; 1% GF is 1% serum supplemented with 4 ng/mL EGF, *n* *=* 15 cells). G1 duration is the time from cytokinesis, visible in phase contrast, to the transition from red to green seen by fluorescence. Migration persistence is calculated during the G1 phase. GF triggers persistent migration and decreases G1 duration. At 1% GF, the distributions of both persistence and G1 duration are scattered. One-way ANOVA followed by Dunnett’s post-Hoc. **c** Migration and cell cycle progression in individual cells at 1% GF. The cells that migrate persistently (with a straight trajectory) appear to have shorter G1 phases than the less persistent ones. G1 duration is indicated in yellow. Scale bar: 30 µm. **d** G1 duration is inversely correlated to migration persistence during G1 (Pearson’s correlation coefficient, *r*, and probability that this correlated distribution arises by chance, *p*, *n* = 39 cells). ARPC1A- and ARPC1B-depleted cells display the same correlated distribution, with ARPC1A-depleted cells exhibiting high persistence and short G1, and with ARPC1B-depleted cells exhibiting low persistence and long G1 (*n* = 20 cells)

To visualise cell cycle progression in live cells, we used the FUCCI system^17^ and established a MCF10A cell line, which expresses two different fluorescent markers for G1 and S-G2, respectively (Fig. 4a). We tracked individual cells over time to obtain simultaneously cell trajectories and cell cycle phases. When cells were starved of growth factors (GF) in a medium without serum, nor EGF, migration persistence dropped and the duration of the G1 phase increased up to 10 h (Fig. 4b). In contrast, when cells were stimulated with the optimal concentration of GF (5% GF, i.e., 5% serum and 20 ng/mL EGF), migration became persistent and G1 lasted only about 5 h. These results suggest an antagonism between G1 duration and migration persistence. When cells were stimulated with 1% GF, distributions of both persistence and G1 duration became greatly scattered in the population. Some cells migrated persistently and appeared to have relatively short G1, whereas less persistent cells appeared to have longer G1 phases (Fig. 4c, Supplementary information, Movie S4). When G1 duration of individual cells was plotted against their migration persistence, a striking linear correlation was revealed. G1 duration was strongly inversely correlated to migration persistence (*r* = −0.88, *P* < 10^−4^; Fig. 4d). Such a correlation with migration persistence was not found for the duration of S and G2 phases (Supplementary information, Fig. S9). We then depleted ARPC1A and ARPC1B in the FUCCI cell line and observed the same scaling relationship, with ARPC1A-depleted cells populating the short G1—high persistence part of the regression line and ARPC1B-depleted cells populating the long G1—low persistence part (Fig. 4d). With or without genetic perturbation, migration persistence, which reflects the polymerisation of cortical branched actin, appears to determine the S-phase entry. But how does cortical branched actin play this role?

### Integration of biochemical and biophysical signals

Cells progress into the cell cycle when they are anchored to the extracellular matrix and stimulated by growth factors.^18^ To investigate whether cortical branched actin transmits these stimuli, we analysed potentiated cells with increased cortical branched actin, through either RAC1 Q61L expression, ARPIN depletion, or ARPC1B overexpression.

**Fig. 5:**
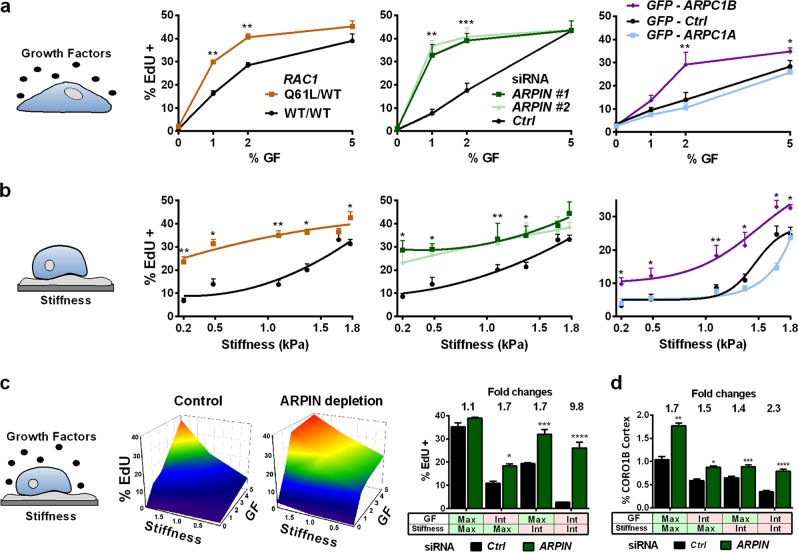
Cortical branched actin integrates mechanical signals with biochemical stimuli. **a** MCF10A cells expressing RAC1 Q61L or GFP-ARPC1B or ARPIN-depleted cells normally stop cycling when deprived of growth factors and cycle at full speed upon full GF stimulation (5%). But they exhibit enhanced cell cycle progression at intermediate levels of stimulation. **b** Cells were plated at the surface of fibronectin-coated gels of various stiffness. On soft substrates, control cells cycle much less than on rigid substrates. Upon RAC1 activation, ARPIN depletion or ARPC1B overexpression, cells cycle more than controls when the substrate is soft. **c** A phase diagram was explored with the four values of GF stimulation and the three values of substratum rigidity. ARPIN depletion expands the surface associated with a high level of cycling. ARPIN depletion exerts the most important effect on cycling, when both stimuli are intermediate (int), instead of maximum (max). **d** Cortical enrichment of CORO1B scales with cell cycle progression. GFP-CORO1B at the cortex was measured as in Fig. 2c (*n* = 15 cells). Data are mean ± s.e.m of technical repeats; one experiment representative of two biological repeats is displayed; Mann–Whitney test for **a** and **b**, two-way ANOVA followed by Sidak’s test for **c** and **d**. When stars are not displayed, no significant difference was found between groups

Growth factors (GFs) tightly regulate cell cycle progression. MCF10A cells cycle at full speed in 5% GF and stop at 0% GF (Fig. 5a). Potentiated cells exhibited the same tight regulation by GFs, with background levels of cycling at 0% GF and maximum cycling at 5% GF. Enhanced cycling was, however, observed at intermediate levels of stimulation (Fig. 5a). Cell adhesion controls cell cycle progression by mechanotransducing the rigidity of the substrate.^19^ We found that more MCF10A cells cycle on rigid substrates with 1.8 kPa stiffness than on softer substrates with 0.2 kPa stiffness (Fig. 5b). At 0.2 kPa, cells display poorly developed lamellipodia, since substrate compliance does not allow them to expand (Supplementary information, Movie S5). More potentiated cells cycled compared with control cells at 0.2 kPa, but not at 1.8 kPa. These two instances show that nucleation of cortical branched actin makes a difference upon suboptimal stimulation, not when cell cycle progression is already fully stimulated by maximum GF stimulation on rigid substrates.

We thus decided to examine the importance of cortical branched actin when combining these two signals, GF and stiffness. In control cells, the tendency of cell cycle progression decays fast, when GF stimulation or substrate stiffness is not optimal (Fig. 5c). In contrast, MCF10A cells with enhanced cortical branched actin cycled about 10 times more than control cells when both GF and rigidity are suboptimal (Fig. 5c; Supplementary information, Fig. S10). Most importantly, cell cycle progression scaled with the enrichment of CORO1B at the cell cortex, measured in a median plane of cells far from potential lamellipodia (Fig. 5d), suggesting that S-phase entry occurred when a sufficiently large amount of ARPC1B containing branches were formed at the cell cortex. The striking combined effect of GF and substratum rigidity is likely physiological, since breast epithelial cells naturally reside in a soft tissue where GF stimulation is limited. But a mammary epithelial cell normally interacts with its neighbouring cells.

**Fig. 6:**
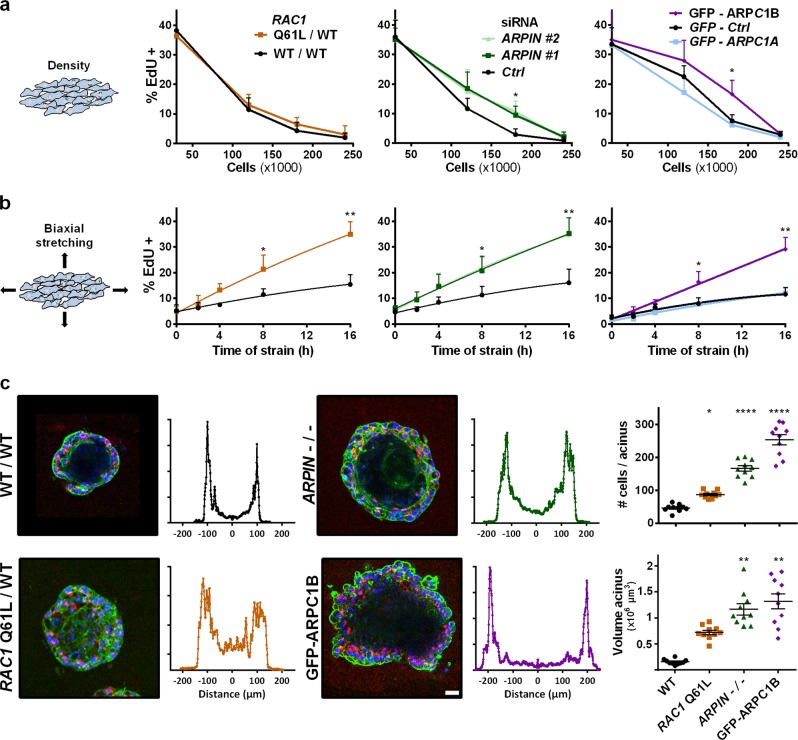
Cortical branched actin potentiates cell cycle progression in epithelial monolayers and in acinus morphogenesis. **a** When more MCF10A cells are plated, cells gradually stop cycling. ARPIN-depleted cells and ARPC1B expressing cells are less sensitive to the cell density-dependent inhibition of cell cycle progression than controls, even if they eventually become fully inhibited, like control cells. **b** Biaxial stretching of a fully confluent monolayer of MCF10A allows cells to resume cell cycle progression. Increased cortical branched actin induced by active RAC1, ARPIN depletion, or ARPC1B overexpression potentiates this mechanotransduced response. **c** Morphogenesis of acini in 3D Matrigel. Acini were stained with DAPI (blue), phalloidin (green) and the apical Golgi marker GM130 (red). Profiles correspond to the sum of radial line scans on the DAPI staining. *ARPIN* knockout or ARPC1B overexpression greatly increases the acinus size. Active *RAC1* also increases the acinus size, but the lumen is not as well defined, because of remaining cells in the center of the structure. Confocal microscopy; Scale bar: 40 µm. Data are mean ± s.e.m of five technical repeats (**a**, **b**) or *n* = 10 (**c**). One experiment representative of two biological repeats is displayed. Mann–Whitney test for **a** and **b**, one-way ANOVA followed by Dunnett’s post-Hoc compared to WT for **c**. When stars are not displayed, no significant difference was found between groups

We thus tested the role of cortical branched actin in density-dependent inhibition of cell cycle progression.^20^ When different number of cells were plated, MCF10A cells cycled in sparse cultures and stopped at confluence. Again, ARPIN-depleted cells or ARPC1B-overexpressing cells kept this regulation, but cycled more than control cells at an intermediate cell density (Fig. 6a). Interestingly, a fully confluent epithelial monolayer can resume cycling when stretched biaxially.^21^ This mechanotransduced response was significantly enhanced in MCF10A cells when cortical branched actin was enhanced (Fig. 6b). Cortical branched actin is, therefore, also involved in mediating density-dependent inhibition of cell cycle progression.

Since branched actin at the cell cortex integrated the three inputs, growth factors, cell-substratum adhesion and cell density, we then tried to induce differentiation of the potentiated cells with more cortical branched actin in a physiological setting. When embedded in matrigel, single MCF10A cells proliferate and differentiate into acini. For this long term assay, which typically requires 3 weeks, we isolated ARPIN knockout cells using the CRISPR-Cas9 system (Supplementary information, Fig. S11). ARPIN-depleted cells and ARPC1B-overexpressing cells formed larger acini containing more cells (Fig. 6c). These acini were composed of correctly polarised cells, as evidenced by the apical localisation of their Golgi complexes, which face the lumen (Supplementary information, Fig. S12). MCF10A cells expressing active RAC1 formed large 3D clusters with less well defined lumen and less polarised cells. All cell lines with enhanced cortical branched actin thus formed 3D structures with an increased number of cells in differentiation conditions. Since unregulated proliferation characterises tumour cells, we sought to examine whether cortical branched actin was also involved in cell transformation.

### Importance for cancer

**Fig. 7:**
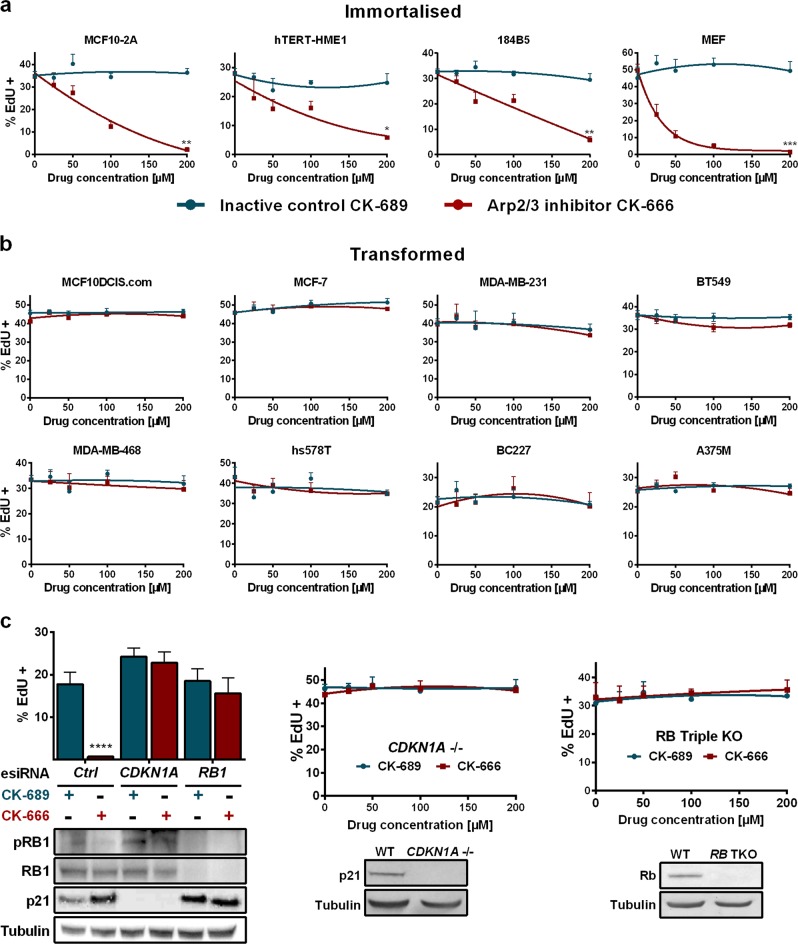
Cell transformation is generally associated with insensitivity to Arp2/3 inhibition. **a** Various immortalised cell lines from human breast were treated with the Arp2/3 inhibitor, CK-666, or with the inactive control, CK-869, at the indicated concentrations. These immortalised human breast cell lines stopped cycling in response to Arp2/3 inhibition, in a dose-dependent manner. Mouse Embryonic Fibroblasts (MEF) behaved the same. **b** Various mammary carcinoma cell lines and the melanoma cell line A375M were insensitive to Arp2/3 inhibition. **c** siRNA mediated inactivation of the tumour suppressor genes *RB1* and *CDKN1A*, which encodes the CDK inhibitory protein p21^WAF1/CIP1^, prevents the cell cycle arrest due to Arp2/3 inhibition. A similar effect is seen in MCF10A cells knock-out for *CDKN1A*,^49^ and mouse embryonic fibroblasts knock-out for all three genes encoding retinoblastoma proteins.^50^ Data are mean ± s.e.m of five technical repeats (**a**–**c**). One experiment representative of 2 biological repeats is displayed, Mann–Whitney test at 200 µM of drug for **a**–**c** (middle and right), twoway ANOVA followed by Sidak’s test for **c** (left). For **b** and **c**, no significant difference between groups was found

MCF10A cells are immortalised, but not transformed, breast epithelial cells. The MCF10DCIS.com cell line is a derivative of MCF10A transformed by oncogenic *HRAS*, which was, furthermore, selected in vivo to give rise to ductal carcinoma in situ.^22^ When MCF10DCIS.com cells were compared to MCF10A cells for their ability to cycle depending on growth factors, substrate rigidity or cell density, they behaved similar to MCF10A cells with enhanced cortical branched actin (Supplementary information, Fig. S13a). The morphogenesis of MCF10DCIS.com in 3D matrigel was strongly altered. Cell clusters grew large, but never developed a lumen (Supplementary information, Fig. S13b). Unlike parental MCF10A cells, MCF10DCIS.com cells cycled even when the Arp2/3 complex was inhibited. We screened 9 additional human breast cell lines and 1 human melanoma cell line for their requirement of cortical branched actin. Strikingly, all three immortalised cell lines stopped cycling in response to Arp2/3 inhibition, like MCF10A cells (Fig. 7a), whereas all 6 transformed ones, i.e., the ones giving rise to tumours upon grafting, continued to cycle upon Arp2/3 inhibition, like MCF10DCIS.com cells (Fig. 7b). Tumour cells activate various oncogenes and inactivate various tumour suppressor genes. We found that inactivating the tumour suppressor gene *RB1* or *CDKN1A*, which encodes the CDK inhibitory protein p21^WAF1/CIP1^, suffices to render the untransformed MCF10A cell line insensitive to Arp2/3 inhibition (Fig. 7c). Together, these results suggest that losing the requirement for branched actin lies at the core of cell transformation.

**Fig. 8:**
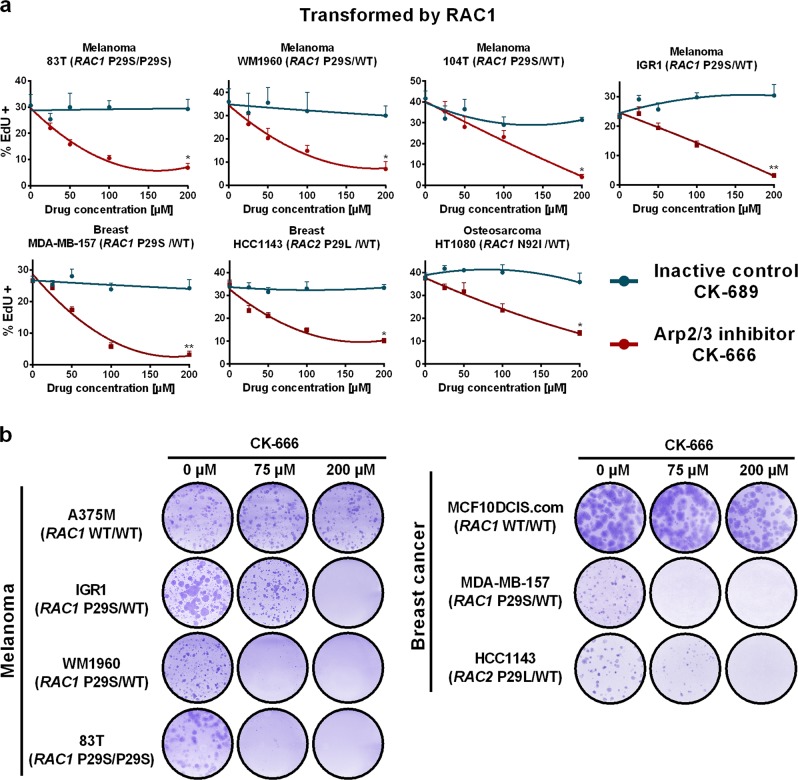
Cancer cell lines transformed by oncogenic RAC1 are sensitive to Arp2/3 inhibition. **a** *RAC1* is an oncogene, which is frequently mutated in melanomas. Four melanoma cell lines transformed by the oncogenic P29S RAC1, mammary carcinoma cell lines, MDA-MB-157 and HCC1143, which carries an equivalent mutation on the *RAC2* gene, and the osteosarcoma cell line, HT1080, which also expresses an active version of RAC1, are all sensitive to Arp2/3 inhibition, unlike the usual tumour cell lines. Data are mean ± s.e.m of 5 technical repeats; one experiment representative of 2 biological repeats is displayed. Mann–Whitney test at 200 µM of drug. **b** The clonogenic growth of melanoma or mammary carcinoma cell lines transformed by RAC proteins is blocked upon Arp2/3 inhibition (two biological repeats)

Since RAC1, upstream of cortical branched actin, is also an oncoprotein activated by the hotspot missense mutation P29S in melanomas,^23^ we collected cell lines that specifically harboured oncogenic forms of RAC proteins.^24–26^ Contrary to randomly picked tumour cell lines, which were insensitive to Arp2/3 inhibition, all the cell lines that contained an active RAC1 protein stopped cycling upon Arp2/3 inhibition (Fig. 8a). Importantly, Arp2/3 inhibition blocked the clonogenic ability of these melanoma and mammary carcinoma cells (Fig. 8b). Oncogenic GTPases of the RAS superfamily are particularly difficult to target, since potential GTPase inhibitors would lock them in the active state just like oncogenic mutations. Our results suggest that RAC1-mediated transformation can be blocked downstream at the level of the Arp2/3 complex, in a manner similar to the block of RAS-mediated transformation exerted by MAP kinase inhibitors.

**Fig. 9:**
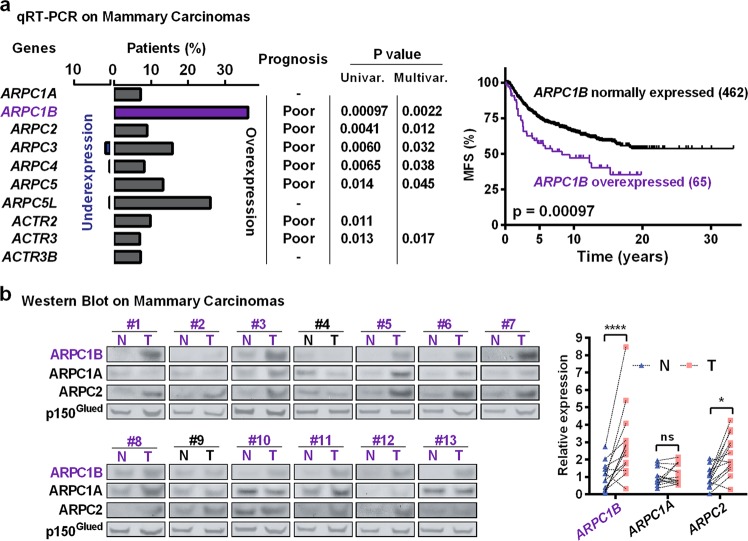
Overexpression of the Arp2/3 complex is associated with a poor prognosis in breast cancer. **a** In a retrospective cohort of 527 breast cancer patients, expression of genes encoding Arp2/3 subunits was measured by qRT-PCR in tumours and healthy tissue. Arp2/3-encoding genes are often overexpressed in mammary carcinomas compared to normal breast. Overexpressions are significantly associated with poor metastasis-free survival (MFS) in 6 genes out of 10 in both uni- and multivariate analyses. Importantly, ARPC1B is the most frequently overexpressed subunit and most significantly associated with poor MFS (Kaplan–Meier survival curve, cut-off at 5). In contrast, the ARPC1A paralogue, is not associated with patient prognosis. **b** Protein expression in mammary carcinomas and normal adjacent tissues was compared by western blot. Overexpression of ARPC1B, but not of ARPC1A, detected in tumours of 11 out of 13 patients, is associated with an overexpression of ARPC2, suggesting that these tumours display elevated levels of Arp2/3 complexes containing ARPC1B. two-way ANOVA followed by Sidak’s test

To examine the role of specific Arp2/3 complexes, we systematically measured the expression of all 10 genes encoding Arp2/3 subunits by qRT-PCR in mammary carcinomas from a retrospective cohort of 527 patients. We compared mRNA levels in tumours to the ones in normal breast. In line with previous reports,^1^ we found that Arp2/3 subunits were globally overexpressed (Fig. 9a). Overexpression of 6 subunits was significantly associated with a poor metastasis-free survival (MFS) of patients in both univariate and multivariate analyses, taking into account other classical prognosis factors (Supplementary information, Fig. S14). *ARPC1B* was the most overexpressed subunit-encoding gene and

most significantly associated with a poor prognosis (Fig. 9a). At 5 years, *ARPC1B* overexpression group had a survival of 57.6% MFS vs. 74.2% for the complementary group expressing normal levels, and at 10 years, MFS dropped to 47.1% vs. 63.9%. *ARPC1B* expression was also related to the Scarff-Bloom-Richardson grade of mammary carcinomas (Supplementary information, Table S1), since 19.9% of grade III patients overexpressed *ARPC1B*, compared to 8.3% for grade II and only 3.3% for grade I. In contrast, the limited overexpression of the paralogous *ARPC1A* gene had no value for the prognosis. We verified by immunohistochemistry that carcinoma cells express the ARPC1B protein (Supplementary information, Fig. S15). Protein extracts were prepared from 13 breast tumours and adjacent healthy tissue. ARPC1B, but not ARPC1A, was overexpressed in a majority of tumours (Fig. 9b). ARPC1B overexpression was associated with ARPC2 overexpression. Together, these results suggest that ARPC1B-containing Arp2/3 complexes are more abundant in mammary carcinomas than in normal tissues and that the enhanced polymerisation of cortical branched actin they provide contributes to breast cancer development.

## Discussion

**Fig. 10:**
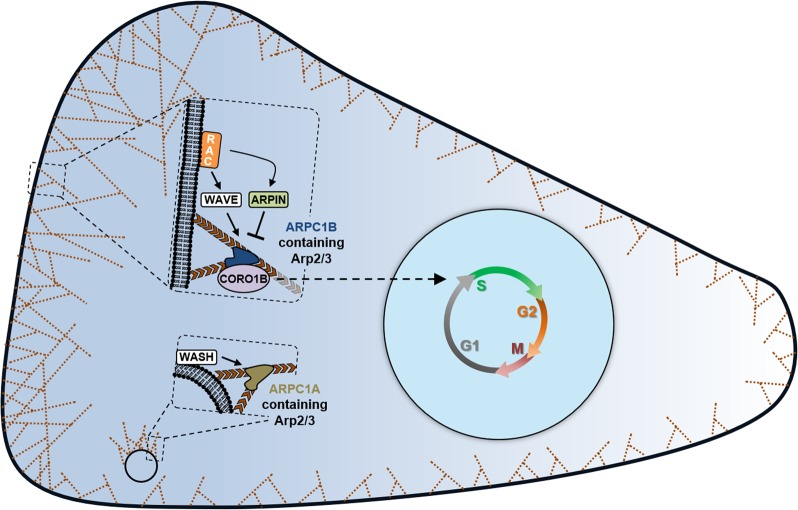
Cortical branched actin controls the G1/S transition. The formation of cortical branched actin by ARPC1B-containing Arp2/3 complexes is under the control of the RAC/WAVE/ARPIN pathway. ARPC1B-containing complexes are specifically sensed by CORO1B, which signals S-phase entry. In contrast, ARPC1A-containing complexes are critical for endosomal branched actin, but do not affect cell cycle progression. Cortical branched actin integrates proliferation signals from soluble growth factors and mechanotransduced adhesion cues

Our work delineates the specificity of the branched actin networks that transduce cell cycle progression. Only the cortical branched actin networks that are specifically nucleated by the Arp2/3 complexes that contain the ARPC1B paralog play this signaling role. This is in contrast with the previously reported centrosomal role of ARPC1B, which is Arp2/3 independent.^27^ Cortical branched actin networks are activated by the WAVE complex—and antagonised by ARPIN—downstream of RAC1 (Fig. 10). They are specifically sensed by CORO1B at the cortex, because type I coronins debranch ARPC1A-dependent actin networks, but stably decorate ARPC1B-containing actin branches, which are immune to debranching.^16^ In optimal conditions, cells stop cycling when branched actin is depleted; in suboptimal conditions, formation of more cortical branched actin by RAC1 activation, ARPIN depletion or ARPC1B overexpression, potentiates cell cycle progression.

We showed that cortical branched actin is the prime integrator of the various inputs that control the G1/S transition. These inputs include both soluble stimuli, such as growth factors, and mechanotransduced cues from cell adhesions. These signals were long known to synergetically control cell cycle progression, despite that the point of integration was not clear.^18^ Similarly, while the actin cytoskeleton, which generates forces, had long been hypothesized to play a role in sensing forces, the elusive forces being sensed have only recently been ascribed to the membrane tension of epithelial cells and to the lamellipodial protrusive activity of single cells.^28,29^ Indeed, branched actin networks were found to respond to mechanical load by becoming denser in vitro,^30^ and lamellipodial actin was also found to become denser when membrane tension was increased.^31^ In this work, we examined the role of various possible actin structures, linear and branched, nucleated by various NPFs, and our data converged on cortical branched actin in line with previous results.

Even if the RAC1-WAVE-Arp2/3 pathway is best known for regulating cell migration, the role of cortical branched actin in transducing cell cycle progression extends beyond the lamellipodial cytoskeleton. The striking anticorrelation between migration persistence and G1 duration only applies to single cells. In epithelial cells, cortical branched actin is well established to play an essential role in the formation of cell-cell junctions.^32,33^ In fact, the RAC1-WAVE-Arp2/3 pathway regulates actin polymerisation all around the cell cortex,^6,7^ and the lamellipodium is just a local and transient amplification of the phenomenon due to positive feedback.^5^ This explains why cortical enrichment of CORO1B in a medial plane of the cell, far from lamellipodia, mirrored cell cycle progression, when growth factor stimulation and substratum rigidity were systematically varied.

Tumour cells are cells that proliferate when they should not. In our work focused on mammary epithelial cells, we found that most tumour cells do not depend on the amount of cortical branched actin to make the decision of S-phase entry. In fact, we were able to render cells insensitive to Arp2/3 inhibition, just by removing a tumour suppressor gene controlling the G1-S transition, such as *RB1* or *CDKN1A*. There are probably many other ways to escape the control exerted by cortical branched actin and the dissection of the downstream signaling pathway that impinges onto the cell cycle machinery is an important task for future studies. However, we reasoned that cells transformed by upstream oncogenes, like *RAC1*, would still require cortical branched actin. This idea was experimentally validated in all cell lines expressing oncogenic forms of *RAC1*. This result is potentially important, as RAC1 oncogenic mutations occur in 5 to 10% of melanoma patients.^34,35^ No targeted therapy is currently available for these patients. Arp2/3 inhibition is thus a potential way to prevent their proliferation and dissemination.

The Arp2/3 complex is overexpressed in many cancers.^1^ In the case of breast cancer, the overexpression of the Arp2/3 complex, of the WAVE complex, or down-regulation of ARPIN expression was associated with poor survival.^36–38^ Given the paralogous specificity of Arp2/3 complexes transducing cell cycle progression, we systematically analysed the deregulation at play in the expression of all Arp2/3 complex subunits in a large cohort of breast cancer patients. ARPC1B was the most overexpressed subunit and the most associated with poor metastasis-free survival, in line with our in vitro results. The multivariate analysis established that ARPC1B levels have a strong prognostic power in isolation, independently from known clinical pathophysiological parameters associated with metastasis-free survival. In addition to a prognostic role, the specificity for ARPC1B-containing Arp2/3 complex uncovered here might provide the basis for future development of small molecule inhibitors, which would specifically target the molecular machinery mediating proliferation and dissemination of tumour cells.

## Material and methods

### Cells and drugs

MCF10A, MCF10DCIS.com, MCF10–2A, 184B5 were grown in DMEM/F12 medium supplemented with 5% horse serum, 20 ng/mL epidermal growth factor, 10 µg/mL insulin, 500 ng/mL hydrocortisone, and 100 ng/mL cholera toxin. Mouse embryonic fibroblasts, MCF7, hs578T, BC227 and HT1080 were grown in DMEM with 10% FBS. MDA-MB-231, MDA-MB-157 and MDA-MB-468 were grown in Leibovitz’s L-15 medium with 10% FBS. hTERT-HME1, BT549, WM1960, A375M, 83 T, 104 T, IGR1 and HCC1143 were grown in RPMI supplemented with 10% FBS. Medium and supplements were from Sigma. Cells were incubated at 37 °C in 5% CO_2_ except cells cultured in Leibovitz’s L-15 medium, which were grown without CO_2_. Primary Mammary Epithelial cells were from Lonza and cultivated in the provided medium. MCF10A *RAC1* Q61L/WT were obtained from Horizon Discovery Ltd. (Cambridge, UK). All cell lines derived from human breast were from the collection of breast cell lines organised by Thierry Dubois (Institut Curie, Paris), A375M, WM1960, IGR1 were from Lionel Larue (Institut Curie, Orsay), 83 T, 104 T were from Yardena Samuels (The Weizmann Institute of Science, Rehovot), MEF triple knockout for Rb were from Julien Sage (Stanford University of Medicine), MCF10A p21^−/−^ were from Ben Ho Park (Johns Hopkins School of Medicine, Baltimore). All cells and stable clones were routinely tested for mycoplasma and found to be negative.

CK-666, CK-689, CK-869, Cytochalasin D, Latrunculin A, and NSC23766 were purchased from Merck, SMIFH2 and Aphidicolin from Sigma. For the clonogenicity assay, medium was replaced with fresh medium containing CK-666 every three days.

### Plasmids, siRNAs, gRNA and transfections

Stable MCF10A cells expressing FUCCI reporters were obtained by sequentially transfecting MCF10A cells, with the ES-FUCCI plasmid (Addgene #6254)^39^ to express mCherry-Cdt1_30–120_ and with the home-made plasmid MXS AAVS1L SA2A Puro bGHpA EF1Flag GFP Geminin Sv40pA AAVS1R to express GFP-Geminin_1–110_ using Lipofectamine 3000 (Thermo Fisher Scientific). Stable MCF10A expressing GFP-CORO1B, GFP-ARPC1A or GFP-ARPC1B were obtained by transfecting MCF10A cells with the plasmid described above. To obtain stable integration of the MXS plasmid at the AAVS1 site, cells were cotransfected with two TALEN plasmids inducing DNA double strand breaks at the AAVS1 locus (Addgene #59025 and 59026).^40^ Cells were selected with 1 µg/mL puromycine and/or 100 µg/mL hygromycine (Invivogen) and pooled.

The MCF10A Arpin knockout cell line, clone #12, was generated with CRISPR/Cas9 system. The targeting sequence GAGAACTGATCGATGTATCT was used to induce the double strand break. Cells were transfected with crRNA:trackRNA duplex and the purified Cas9 protein by Lipofectamine CRISPRMAX™ Cas9 Transfection Reagent (Thermofisher Scientific). The next day, cells were subjected to dilution at 0.8 cells/well in 96 well plate. Single clones were expanded and analyzed by ARPIN western blot. A frameshift at both alleles in positive clones was confirmed by sequencing.

To perform screens of genes involved in the cortical branched actin pathway, genes were knocked down with esiRNAs (Sigma), which correspond to long dsRNAs processed in vitro into a collection of short siRNAs. Because they cover a large part of the gene, esiRNAs are more consistently efficient at depleting mRNAs than individual siRNA sequences. An esiRNA corresponding to GFP was used as a control. Only ARPIN and BRK1 were targeted by individual siRNAs (ON-TARGETplus, Dharmacon): ARPIN#1 GUGGAUGUAUCUCGGCACA, ARPIN#2 AUGGCAGGAAGGAGCGCUA, and BRK1#1 GGGCUAACCGGGAGUACAUUU, BRK1#2 CGAUAUGUCUUGUCGUUCAUU, BRK1#3 ACACUAAACGAGAAAUUGAUU, BRK1#4 GAACGGAGAAUAGAGUACAUU. In this case, the control siRNA was the non-targeting siRNA designed by Dharmacon. MCF10A cells were transfected using lipofectamine RNAiMax according to the recommended reverse transfection protocol (Thermo Fisher Scientific) and analysed 72 h later. To knock-down the Arp2/3 complex, cells were retransfected the next day with the same quantity of esiRNA using the forward transfection protocol and were analysed 96 h after the first transfection.

### Antibodies, western blot and immunofluorescence

Home-made rabbit polyclonal antibodies were previously described: anti-ARPIN,^4^ anti-CCDC53,^13^ anti-BRK1,^41^ anti-Sra1, anti-Nap1, anti-Wave2, and anti-Abi1.^42^ Antibodies targeting RB1 (#9309) and p21 (#2947) were from Cell Signaling, Arp3 (#07–272), ARPC2 (#07–227) and Cortactin (#05–180) from Millipore, CEP55 (sc-374051), VPS35 (#Sc-374372), ARPC1B (#Sc-137202 for IF of Fig. 3f) and Coronin1B (#Sc-66838) from Santa-Cruz Biotechnology, ARPC1A (#HPA004334), ARPC1B (#HPA004832 for WB) and Coronin1B (#WH0057175M1) from Sigma, GM130 (#610823) and p150Glued (#610473) from BD Biosciences, Acetyl Tubulin (C3B9-hFc) from the TAb-IP recombinant antibodies platform of Institut Curie, Paris, France and Tubulin (Clone E7) from Developmental Studies Hybridoma Bank.

MCF10A cells were lysed in RIPA buffer and analysed by western blot. SDS-PAGE was performed using NuPAGE 4–12% Bis-Tris gels (Life Technologies). Nitrocellulose membranes were developed with horseradish peroxidase (HRP)-coupled antibodies (Sigma) and SuperSignal West Pico chemiluminescent substrate (Thermo Fisher Scientific) or with alkaline phosphatase-coupled antibodies (Promega) and NBT-BCIP as substrates (Promega).

For immunofluorescence, cells were fixed with 4% paraformaldehyde and permeabilised with 0.5 % Triton X-100. Quantification was performed on at least 2 different biological replicates by measuring signal intensity with ImageJ. Endosomal branched actin and cortical CORO1B were imaged using a confocal laser scanning microscope (TCS SP8, Leica) equipped with an inverted frame (Leica), a high NA oil immersion objective (HC PL APO 63×/1.40, Leica) and a white light laser (WLL, Leica). Radial line scans were drawn as described.^4^ To measure endosomal enrichment of the protein of interest, images were thresholded using the endosomal VPS35 signal to create a Region Of Interest (ROI), then the mean intensity of the staining was measured in this ROI. Endosomal enrichment was defined as the ratio of the mean intensity in the ROI divided by the mean intensity in the whole cell. To measure CORO1B at the cortex, CellMask Deep Red Plasma membrane Stain (Thermo Fisher Scientific) used as described^43^ defined cell periphery. Then the integrated intensity of GFP-CORO1B in the cortical region was divided by the integrated intensity throughout the cell. To count the number of cells displaying lamellipodia, a lamellipodium was defined as a convex membrane staining of Cortactin wider than 2 µm.

### Cell cycle assays

For EdU assays, coverslips were coated with 10 µg/mL bovine fibronectin (Sigma) for 30 min at 37 °C in PBS. 15,000 cells per cm² were plated onto the coverslips. Drug treatment was for 16 h. EdU (Thermo Fisher Scientific) was added to cells 1 h before fixation. Cells were processed as for immunofluorescence but labelled with the Alexa Fluor 488 Click-iT EdU Imaging Kit (Thermo Fisher Scientific, #C10337) and DAPI (Thermo Fisher Scientific). For EdU quantification, the percentage of cells in S-phase was scored as the ratio of EdU-positive nuclei / DAPI-stained nuclei in segmented images (code available upon request). For each condition, at least 200 cells were counted. For FACS analysis, cells were trypsinised before fixation and the percentage of cells in the different phases of the cell cycle was analysed on a Guava easyCyte system (Millipore). β-galactosidase activity was used to stain senescent cells after 4 days of treatment (Senescence Cells Histochemical Staining Kit, Sigma).

For GF stimulation, cells were starved of serum and EGF for 36 h and then stimulated for 16 h. 1% GF corresponds to 1% Horse serum and 4 ng/mL of EGF. For the control of substrate rigidity, polyacrylamide gels of 0.2 kPa, 1.1 kPa and 1.78 kPa were prepared as described.^44^ To analyse density dependence, the indicated number of cells was seeded on fibronectin-coated coverslips 48 h before EdU incorporation. To stretch epithelial monolayers, 10^6^ cells were plated on pronectin-coated flexible PDMS substrates (BioFlex culture plates) 36 h before a biaxial stretch of 18% was applied for 16 h with the FX-5000 Tension System (FlexCell International Corporation). For the clonogenicity assay, cells were seeded in 6-well dishes, grown for 3 weeks, fixed and stained with crystal violet. For the acinus assay, single cells were seeded on a 1 mm thick solidified layer of matrigel (growth factor reduced, Thermo Fisher Scientific, #CB-40230C) and grown for 21 days in MCF10A medium with 2% horse serum and 5 ng/mL of EGF, as previously described.^45^

### Videomicroscopy and analysis of cell migration

Videomicroscopy was performed on an inverted Axio Observer microscope (Zeiss) equipped with a Pecon Zeiss incubator XL multi S1 RED LS (Heating Unit XL S, Temp module, CO_2_ module, Heating Insert PS and CO_2_ cover), a definite focus module and a Hamamatsu camera C10600 Orca-R2. Pictures were taken every 5 min for 24 h for migration of MCF10A and for 48 h for MCF10A-FUCCI using the Plan-Apochromat 20×/0.80 air objective. In the single cell migration assay, migrating cells making cell contacts for more than 4 h during the whole tracking period were excluded from the analysis. Random migration was analysed with ImageJ and DiPer as described.^46^ Single cell persistence corresponds to the area under the curve of the directional autocorrelation function (without gap) plotted with DiPer.

### Patient cohort for mRNA analysis

All patients (mean age 62 years, range 29–91 years) met the following criteria: primary unilateral nonmetastatic breast carcinoma for which complete clinical, histological and biological data were available; no radiotherapy or chemotherapy before surgery; and full follow-up at Institut Curie - Hospital René Huguenin. All patients before 2007 were informed that their tumour samples might be used for scientific purposes and had the opportunity to decline. Since 2007, patients treated in our institution have given their approval by signed informed consent. This study was approved by the local ethics committee (Breast Group of René Huguenin Hospital). Treatment (information available for 524 patients) consisted of modified radical mastectomy in 320 cases (61%) or breast-conserving surgery plus locoregional radiotherapy in 204 cases (39%). The patients had a physical examination and routine chest radiotherapy every 3 months for 2 years, then annually. Mammograms were done annually. Adjuvant therapy was administered to 416 patients, consisting of chemotherapy alone in 130 cases, hormone therapy alone in 178 cases and both treatments in 108 cases. During a median follow-up of 10.5 years (range 1 month to 36.3 years), 210 patients developed metastasis. Sixteen specimens of adjacent normal breast tissue from breast cancer patients and normal breast tissue from women undergoing cosmetic breast surgery were used as sources of normal RNA.

### qRT-PCR

Specific mRNAs were quantified from the cycle number (Ct value) at which the increase in the fluorescence signal started to be detected by the laser detector of the ABI Prism 7900 sequence detection system (Perkin-Elmer Applied Biosystems, Foster City, CA) as previously described.^47^ Specific transcripts were quantified using the following primers: ARPC1A-U (5′-ACTCAAGGAGCACAACGGACAC-3′) and ARPC1A-L (5′-GGTCTGCCCCACAAGTGACA-3′) for ARPC1A gene (PCR product of 80 bp); ARPC1B-U (5′-CACAAGAACAGCGTCAGCCAGAT-3′) and ARPC1B-L (5′-CTCCAAGCTCTTCACATCCCAGATA-3′) for ARPC1B gene (PCR product of 117 bp); ARPC2-U (5′-CGTCTGACTTCCTCAAGGTGCTG-3′) and ARPC2-L (5′-GATGAAAACGTCTTCCCCGTGAT-3′) for ARPC2 gene (PCR product of 88 bp); ARPC3-U (5′-ACTTCAAGGCCAATGTCTTCTTCA-3′) and ARPC3-L (5′-GCTTTTGGAATTGCACTTTTGC-3′) for ARPC3 gene (PCR product of 125 bp); ARPC4-U (5′-TTGCTGTGAAACAGGCTGATGAG-3′) and ARPC4-L (5′-AGAAAGCTGATATCATACCCCTCCA-3′) for ARPC4 gene (PCR product of 130 bp); ARPC5-U (5′-GCAATGGCATGAAAAGGCACT-3′) and ARPC5-L (5′-ATCCACTTCCTGCCAGACTACACA-3′) for ARPC5 gene (PCR product of 96 bp); ARPC5L-U (5′-CATGCAGCCTTGCGGAACTCT-3′) and ARPC5L-L (5′-CCCTGGGCTCGCTCCTTCA-3′) for ARPC5L gene (PCR product of 65 bp); ACTR2-U (5′-AGAACGAGTTTTGAAGGGTGATGT-3′) and ACTR2-L (5’-TAGAACTGCACCACCCAGGAAT-3′) for ACTR2 gene (PCR product of 106 bp); ACTR3-U (5′-CGGGGTATACAAAACTAGGATATGCT-3′) and ACTR3-L (5’-CATCACCCTCCTTTGAGCTTGA-3′) for ACTR3 gene (PCR product of 116 bp); ACTR3B-U (5′-CTTGGCTACGCAGGCAACACT-3′) and ACTR3B-L (5′-ACTCCCCTCAACACTCTCCTTTGA-3′) for ACTR3B gene (PCR product of 110 bp); TBP-U (5′-TGCACAGGAGCCAAGAGTGAA-3′) and TBP-L (5′-CACATCACAGCTCCCCACCA-3′) for the TBP gene (PCR product of 132 bp), which was the reference gene used for normalisation.

### Patient biopsies for western blot

20 patients with invasive breast carcinoma (T_1–3_N_0–3_M_0–1_; grade 1–2; luminal A, B, and HER2^+^), between 46 and 68 years of age (mean age: 55.2 ± 9.0), and treated in the Tomsk National Research Medical Center between 2013 and 2015 were included. All cases were without any preoperative therapy and underwent mastectomy, radical or sectoral resection. The procedures followed in this study were in accordance with the Helsinki Declaration (1964, amended in 1975 and 1983). This study was approved by the institutional review board; all patients signed an informed consent for voluntary participation.

For tissue lysis, samples were first weighted. 3.75 µL of cold PB buffer (100 mM HEPES, 300 mM NaCl, pH 7.7) supplemented with a mix of protease inhibitors (1 µg/mL Leupeptin, 80 µg/mL Antipain, 1 mM PMSF, 80 µg/mL Chymostatine, 500 µg/mL Pefabloc and 240 µg/mL benzamidine) were added to each mg of tissue. Samples were then homogenised at 4 °C using a manual piston. After homogenisation, the same volume of concentrated RIPA buffer (100 mM HEPES, 300 mM NaCl, 2% NP-40, 1% Sodium Deoxycholate, 0.2% SDS, pH 7.7) supplemented with protease inhibitors, was added and incubated on ice for 30 min. The extract was clarified by centrifugation at maximum speed for 10 min in a tabletop centrifuge at 4 °C. Total protein concentration of the clarified lysate was determined using the BCA assay (Thermoscientific). Ten micrograms of samples was fractionated by SDS-PAGE and revealed by western blotting. Signals were quantified by densitometry using ImageJ. The ratio of specific signals to the signal of the p150Glued loading control is calculated and normalised to the mean ratio for normal tissue.

### Biostatistics

Statistical analysis of the results was carried out with GraphPad software. When data satisfied the two criteria of normality and equal variance, parametric tests were used: t-test to compare two groups; ANOVA for more than two, followed by Dunnett’s post-Hoc test or by Sidak’s test. When data did not satisfy both criteria, non-parametric tests were applied: Mann–Whitney to compare two groups; Kruskal–Wallis for more than two. Except when indicated otherwise, a representative experiment is plotted and results are expressed as mean ± s.e.m of a minimum of five technical repeats. Differences were considered significant at confidence levels greater than 95% (*P* < 0.05). Four levels of statistical significance are distinguished: *P < 0.05; **P < 0.01; ***P < 0.001; ****P < 0.0001.

In patient samples, the distribution of mRNA levels was characterised by median values and ranges. Relationships between mRNA levels and clinical parameters were identified using non-parametric tests, namely the Mann–Whitney *U* test and the Kruskal–Wallis test. Metastasis-free survival (MFS) was determined as the interval between initial diagnosis and detection of the first metastasis. Data were summarised in a receiver operating characteristic (ROC) curve.^48^ The area under the curve was calculated as a single measure to discriminate efficacy. Survival distributions were estimated by the Kaplan–Meier method, and the significance of differences between survival rates was ascertained with the log-rank test. Cox’s proportional hazard regression model was used to assess prognostic significance in multivariate analysis.

## Supplementary information

Supplementary information, Movie S1

Supplementary information, Movie S2

Supplementary information, Movie S3

Supplementary information, Movie S4

Supplementary information, Movie S5

Supplementary information, Movie legends

Supplementary FigureS1

Supplementary FigureS2

Supplementary FigureS3

Supplementary FigureS4

Supplementary FigureS5

Supplementary FigureS6

Supplementary FigureS7

Supplementary FigureS8

Supplementary FigureS9

Supplementary FigureS10

Supplementary FigureS11

Supplementary FigureS12

Supplementary FigureS13

Supplementary FigureS14

Supplementary FigureS15

Supplementary TableS1
