# Supplementary material for: Cortical branched actin determines cell cycle progression
Source: Cell Res. 2019 Apr 10;29(6):432–45. doi: 10.1038/s41422-019-0160-9 (PMC6796858; doi:10.1038/s41422-019-0160-9)
Supplement: Supplementary file 6 — Supplementary information, Movie legends [file 41422_2019_160_MOESM6_ESM.pdf]

Supplementary movie legends

Video S1: Arp2/3 inhibition does not affect mitosis. FUCCI cells (described in Fig.4) were submitted to Arp2/3 inhibition using CK-666 or treated with the inactive compound, CK-689. 3 examples of mitosis are displayed in both cases. Movies were registered with respect to cytokinesis. CK-666 clearly affects membrane protrusions, but not cytokinesis, which relies on actomyosin contraction. Such movies were used to calculate mitosis duration (relates to Fig. S3D). Scale bar : 30 µm.

Video S2: Contribution of Arp2/3 subunits to the migration persistence of MCF10A cells. A representative cell for each condition is shown and tracked (relates to Fig. 3D). Scale bar : 30 µm.

Video S3: Effect of ARPC1A or ARPC1B overexpression on the migration persistence of MCF10A cells. A representative cell for each condition is shown and tracked (relates to Fig. 3I). Scale bar : 30 µm.

Video S4: FUCCI cells tracked during the G1 phase. Red cells are tracked from the end of mitosis to S-phase entry indicated by the shift to the yellow color (relates to Fig.4C). Three examples of cells illustrate the inverse relationship between migration persistence in G1 (red track) and the duration of the G1 phase (written in yellow). Scale bar : 30 µm.

Video S5: Videomicroscopy of cells plated on soft and stiff substrates. On soft substrates (0.2 kPa), MCF10A cells do not form lamellipodia, are poorly spread and do not migrate. On stiff substrates (1.8 kPa), cells form lamellipodia, spread and migrate. This behavior correlates with cell cycle progression (relates to Fig.5B). Scale bar : 20 µm.
