# Supplementary material for: Cortical branched actin determines cell cycle progression
Source: Cell Res. 2019 Apr 10;29(6):432–45. doi: 10.1038/s41422-019-0160-9 (PMC6796858; doi:10.1038/s41422-019-0160-9)
Supplement: Supplementary file 7 — Supplementary FigureS1 [file 41422_2019_160_MOESM7_ESM.pdf]

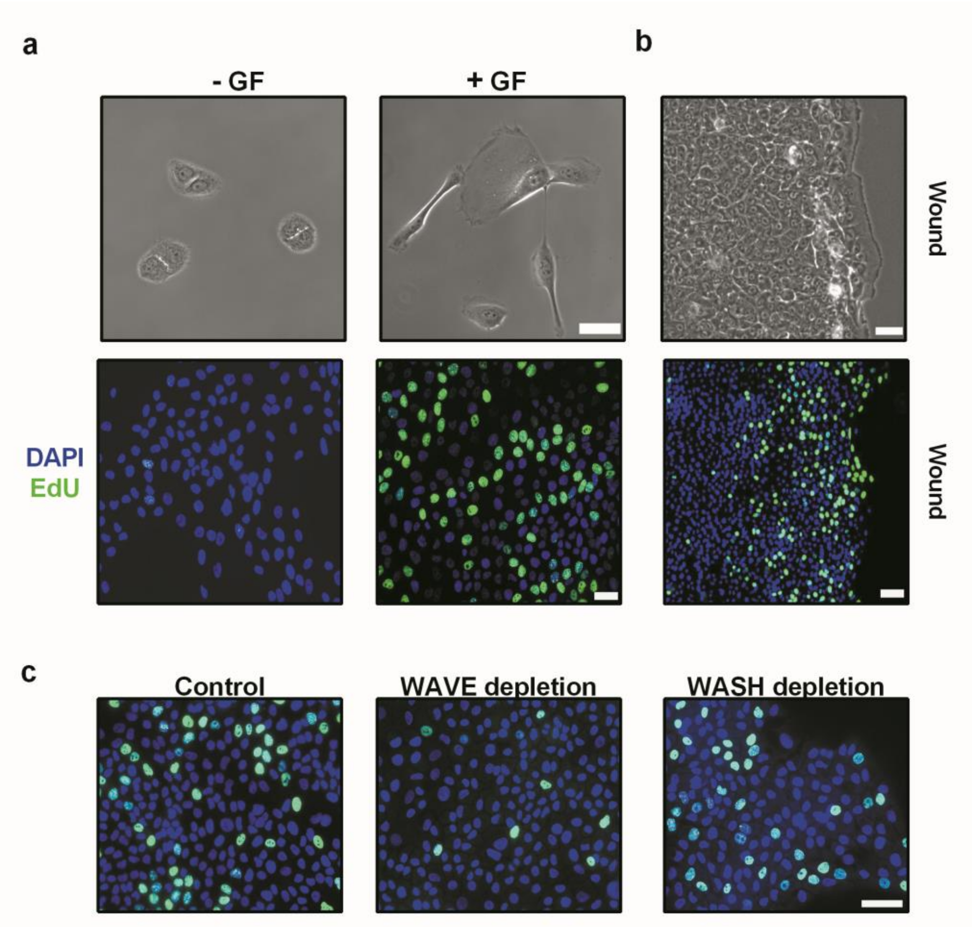

Figure S1: Situations where lamellipodium formation appears coupled to cell cycle progression. a MCF10A cells were deprived of serum and EGF for 36 h (-GF) and then stimulated for 16 h with 5 % serum and 20 ng/ml EGF (+GF). GF treatment induces lamellipodium formation (phase contrast, left panel) and cell cycle progression (EdU incorporation, right panels). **b** Confluent MCF10A cells were subjected to wounding. After 6 h, cells at the border of the wound develop lamellipodia. This mechanical induction of lamellipodia is accompanied by cell cycle progression in the first rows of cells facing the wound. **c** WAVE complex depletion (esi*BRK1*) that prevents lamellipodium formation blocks cell cycle progression, whereas WASH depletion (esi*CCDC53*) that impairs endosomal trafficking does not. Scale bars: 40µm.
