# Supplementary material for: Cortical branched actin determines cell cycle progression
Source: Cell Res. 2019 Apr 10;29(6):432–45. doi: 10.1038/s41422-019-0160-9 (PMC6796858; doi:10.1038/s41422-019-0160-9)
Supplement: Supplementary file 8 — Supplementary FigureS2 [file 41422_2019_160_MOESM8_ESM.pdf]

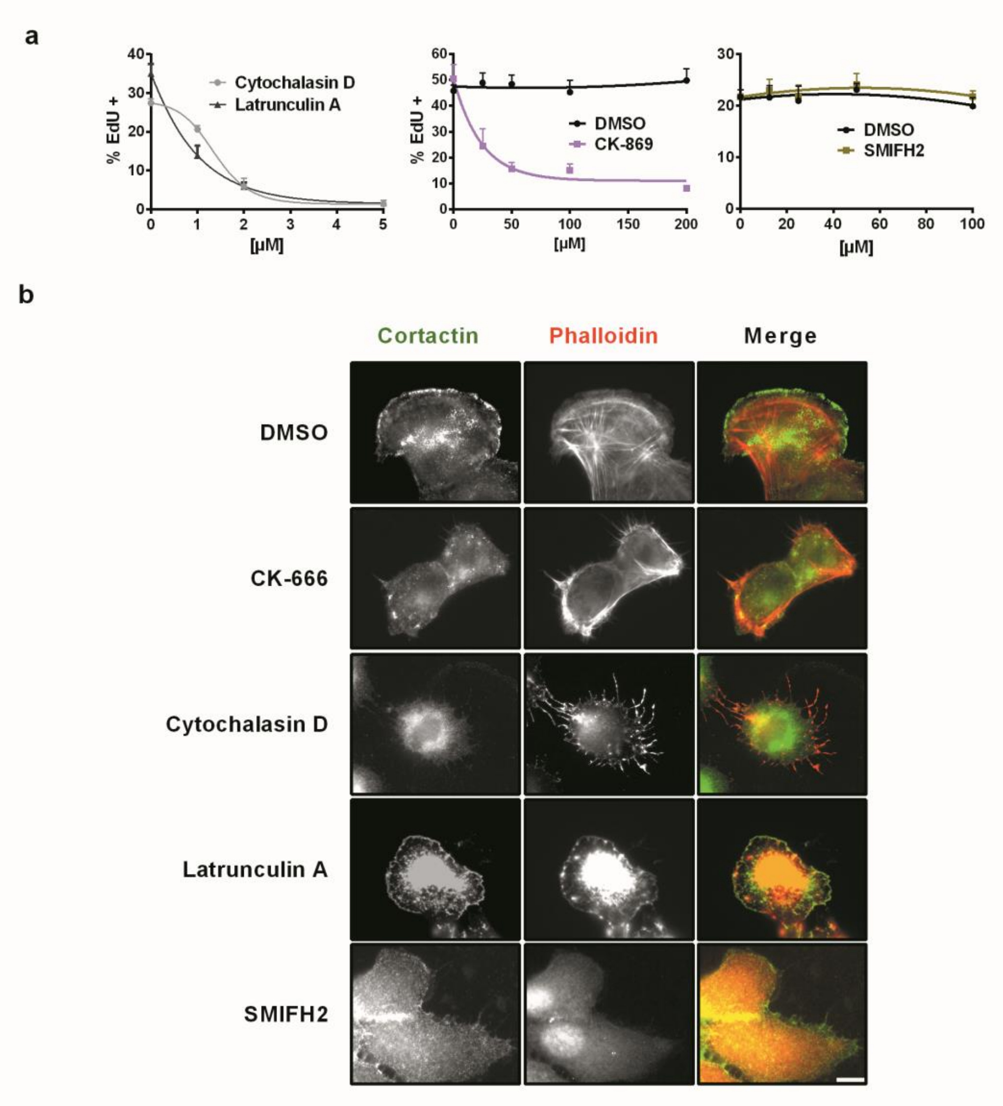


**Figure S2: Branched actin is required for S-phase entry of MCF10A cells**. **a** The two actin drugs, Cytochalasin D, which impairs filament elongation, and Latrunculin A, which sequesters monomeric actin, block cell cycle progression in a dose-dependent manner. The Arp2/3 inhibitor CK-869 blocks cell cycle progression in a dose-dependent manner, whereas the general formin inhibitor SMIFH2 has no effect. **b** Drug treatments were verified to affect cell morphology and the actin cytoskeleton. Cells were stained with phalloidin to detect actin filaments and Cortactin antibodies to detect branched actin networks. Treatments with 2 µM Cytochalasin D and 2 µM Latrunculin A induce cell retraction. Treatment with 100 µM CK-666 inhibits the formation of lamellipodia, best revealed by Cortactin staining. Treatment with 50 µM SMIFH2 inhibits the formation of stress fibers, as seen by phalloidin staining. All treatments were for 4 h. Scale bar : 10 µm.
