# Supplementary material for: Cortical branched actin determines cell cycle progression
Source: Cell Res. 2019 Apr 10;29(6):432–45. doi: 10.1038/s41422-019-0160-9 (PMC6796858; doi:10.1038/s41422-019-0160-9)
Supplement: Supplementary file 9 — Supplementary FigureS3 [file 41422_2019_160_MOESM9_ESM.pdf]

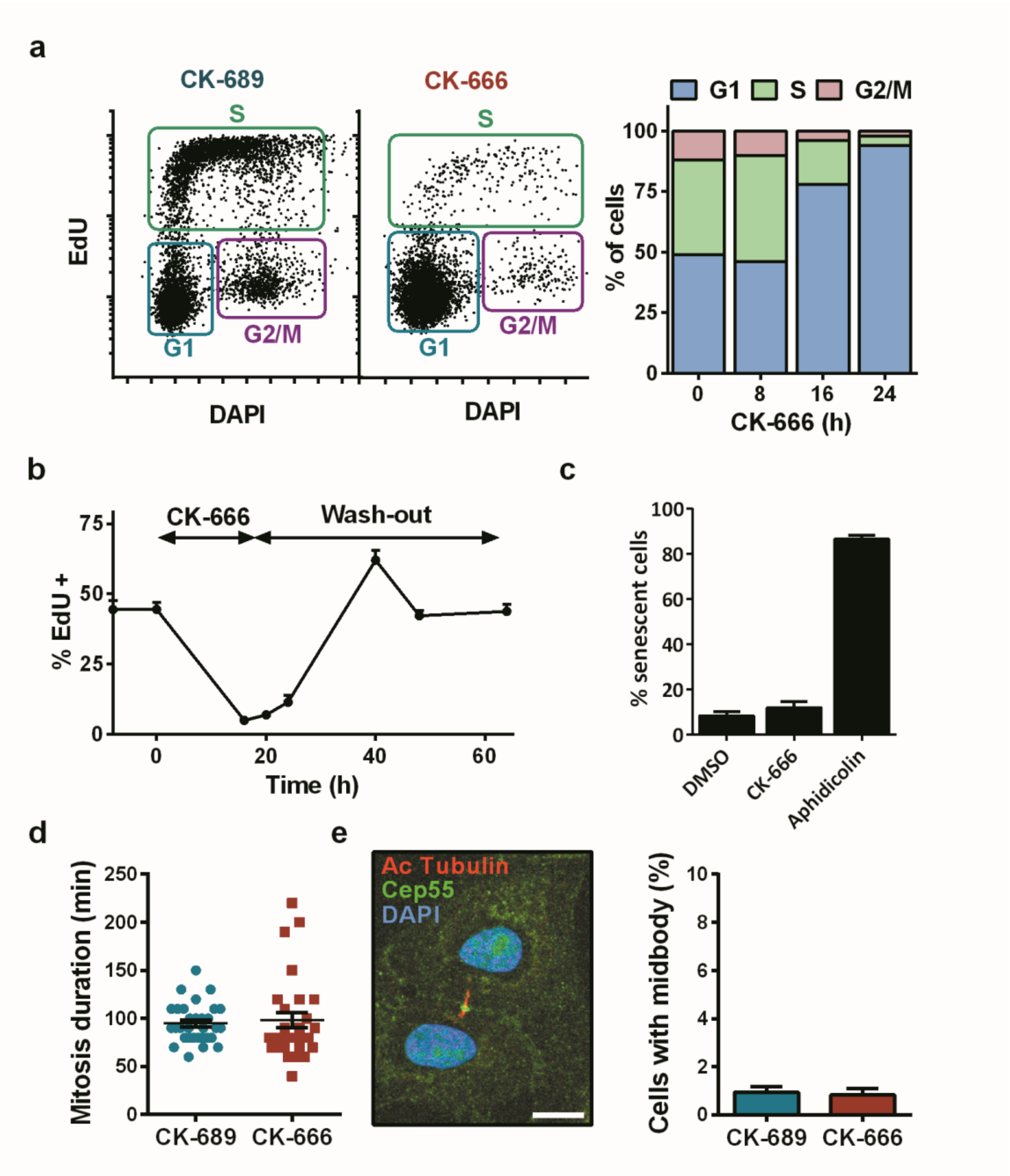

Figure S3: Branched actin is required in G1 for S-phase entry of MCF10A cells. **a** MCF10A cells accumulate in G1 upon Arp2/3 inhibition (FACS). **b** The cell cycle block induced by CK-666 is reversible. The overshoot upon drug washout is due to the synchronisation associated with G1 release. **c** Unlike Aphidicolin, CK-666 does not induce cellular senescence. **d** Arp2/3 inhibition does not affect the duration of mitosis. **e** Arp2/3 inhibition does not affect cytokinesis, as the number of cells with a midbody, labelled with Cep55 and acetylated Tubulin, does not change upon CK-666 treatment. Confocal microscopy, scale bar : 10 µm.
