# Supplementary material for: Cortical branched actin determines cell cycle progression
Source: Cell Res. 2019 Apr 10;29(6):432–45. doi: 10.1038/s41422-019-0160-9 (PMC6796858; doi:10.1038/s41422-019-0160-9)
Supplement: Supplementary file 10 — Supplementary FigureS4 [file 41422_2019_160_MOESM10_ESM.pdf]

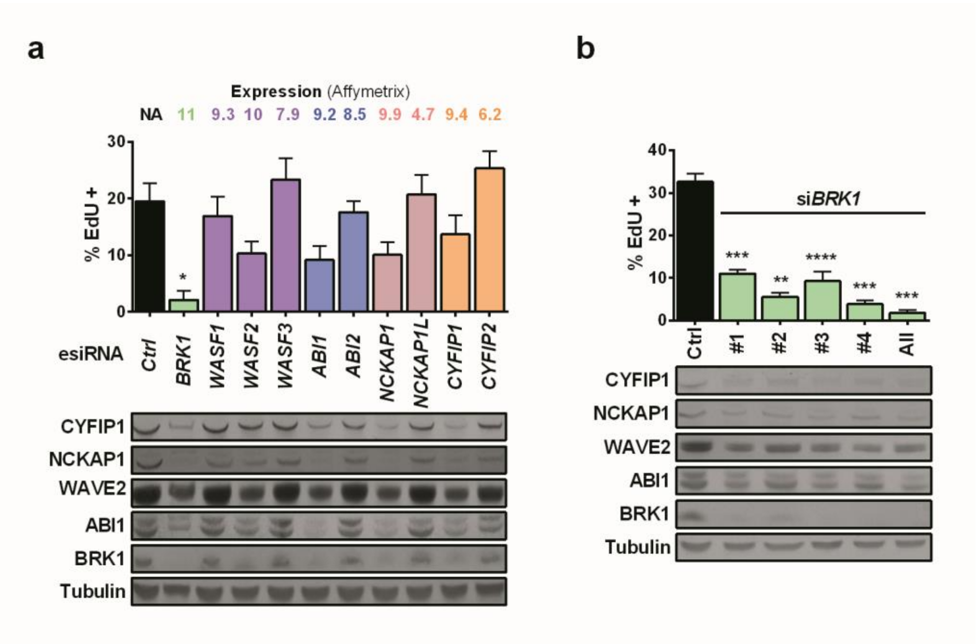

Figure S4: Branched actin at the lamellipodia is specifically required for S-phase entry of MCF10A cells. **a** Depletion of WAVE complex subunits does not suggest a specificity for particular paralogs. For each subunit, only the most expressed paralog impairs cell cycle progression when depleted (Affymetrix expression in non-linear arbitrary units). Western blot analysis of the most abundant ubiquitous WAVE complex[42] in response to subunit depletion. **b** Validation of the use of BRK1 siRNA to deplete the WAVE complex. Four individual siRNA sequences efficiently deplete the WAVE complex and impair cell cycle progression.
