# Supplementary material for: Cortical branched actin determines cell cycle progression
Source: Cell Res. 2019 Apr 10;29(6):432–45. doi: 10.1038/s41422-019-0160-9 (PMC6796858; doi:10.1038/s41422-019-0160-9)
Supplement: Supplementary file 11 — Supplementary FigureS5 [file 41422_2019_160_MOESM11_ESM.pdf]

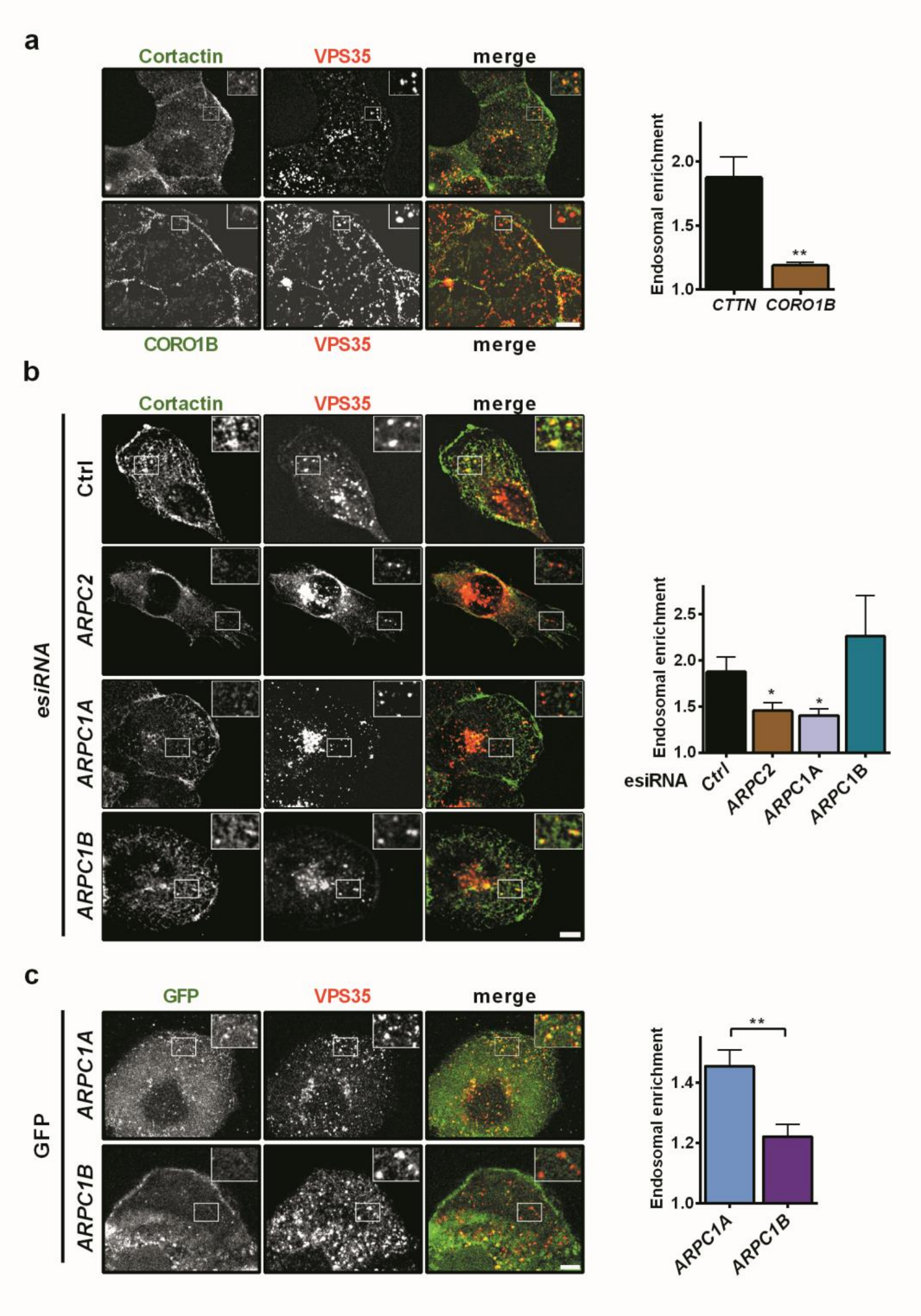

Figure S5: CORO1B and ARPC1B do not localise at the surface of endosomes. a CORO1B is not associated with VPS35 containing retromer domains, which recruit the WASH complex. **b** Depletion of Arp2/3 complexes containing ARPC1A, but not ARPC1B, impairs the formation of endosomal branched actin associated with VPS35. **C** GFP-ARPC1A, but not GFP-ARPC1B, is enriched at endosomes. Confocal microscopy, scale bar : 5 µm.
