# Supplementary material for: Cortical branched actin determines cell cycle progression
Source: Cell Res. 2019 Apr 10;29(6):432–45. doi: 10.1038/s41422-019-0160-9 (PMC6796858; doi:10.1038/s41422-019-0160-9)
Supplement: Supplementary file 12 — Supplementary FigureS6 [file 41422_2019_160_MOESM12_ESM.pdf]

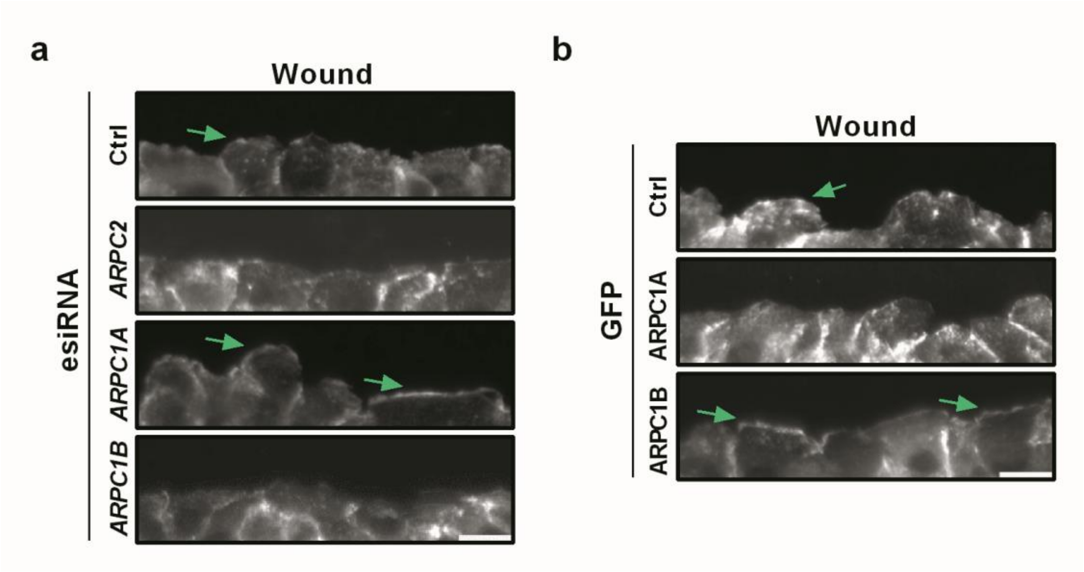

Figure S6: Wound healing enriches ARPC1B-containing Arp2/3 complexes at the edge. Enrichment of Arp2/3 complexes at the leading edge was revealed by Cortactin staining and indicated by green arrows. The enrichment is more obvious when ARPC1A is depleted by esiRNA (A) or when ARPC1B is overexpressed (B). Scale bar : 20 µm.
