# Supplementary material for: Cortical branched actin determines cell cycle progression
Source: Cell Res. 2019 Apr 10;29(6):432–45. doi: 10.1038/s41422-019-0160-9 (PMC6796858; doi:10.1038/s41422-019-0160-9)
Supplement: Supplementary file 13 — Supplementary FigureS7 [file 41422_2019_160_MOESM13_ESM.pdf]

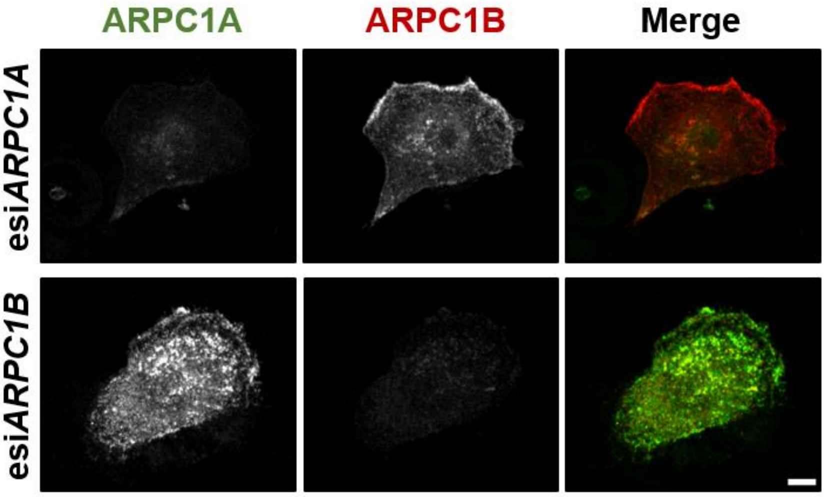

Figure S7: ARPC1A and ARPC1B antibodies are specific. The staining of ARPC1A and of ARPC1B is lost upon esiRNA mediated depletion of their respective target protein. Confocal microscopy, scale bar : 5 µm. Relates to Fig. 3f.
