# Supplementary material for: Cortical branched actin determines cell cycle progression
Source: Cell Res. 2019 Apr 10;29(6):432–45. doi: 10.1038/s41422-019-0160-9 (PMC6796858; doi:10.1038/s41422-019-0160-9)
Supplement: Supplementary file 14 — Supplementary FigureS8 [file 41422_2019_160_MOESM14_ESM.pdf]

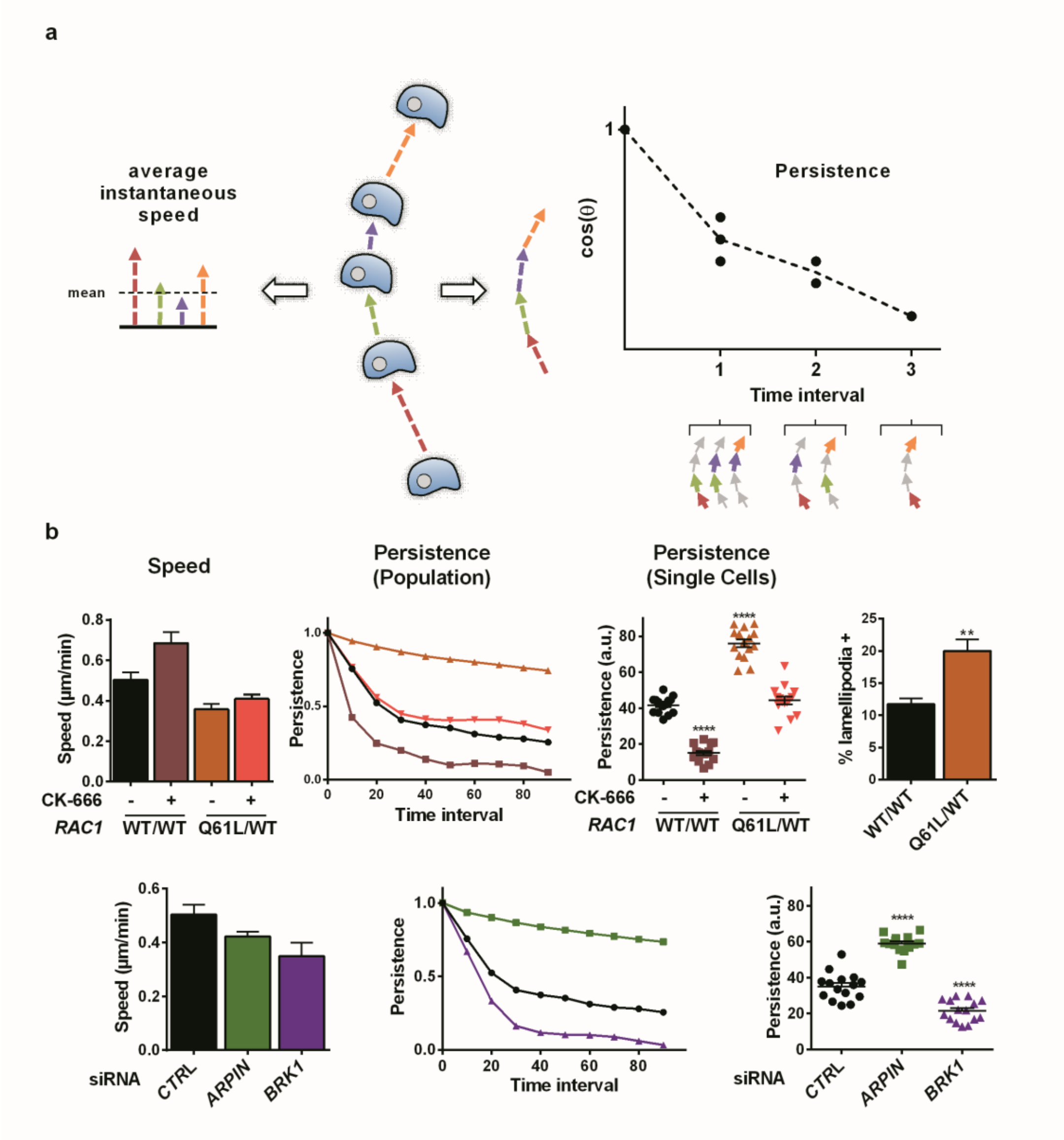

Figure S8: Arp2/3 activity controls migration persistence, not speed. a Scheme explaining how instantaneous speed and persistence are calculated from cell trajectories. These two parameters reflect the amplitude and the orientation of displacement vectors, respectively. Persistence is an index of the maintenance of direction over time. It is calculated using an autocorrelated function[46]. **b** The activity of the Arp2/3 complex is required for migration persistence, not for speed. RAC1 Q61L increases migration persistence and lamellipodia formation in an Arp2/3-dependent manner but not speed of migration. Depletion of the WAVE complex through siRNAs targeting BRK1 impairs migration persistence, whereas depletion of ARPIN increases migration persistence. Persistence can be plotted for the whole cell population at once, or for each single cell, by measuring the area under the curve of each autocorrelation function. It is then expressed in arbitrary units (a.u.).
