# Supplementary material for: Cortical branched actin determines cell cycle progression
Source: Cell Res. 2019 Apr 10;29(6):432–45. doi: 10.1038/s41422-019-0160-9 (PMC6796858; doi:10.1038/s41422-019-0160-9)
Supplement: Supplementary file 15 — Supplementary FigureS9 [file 41422_2019_160_MOESM15_ESM.pdf]

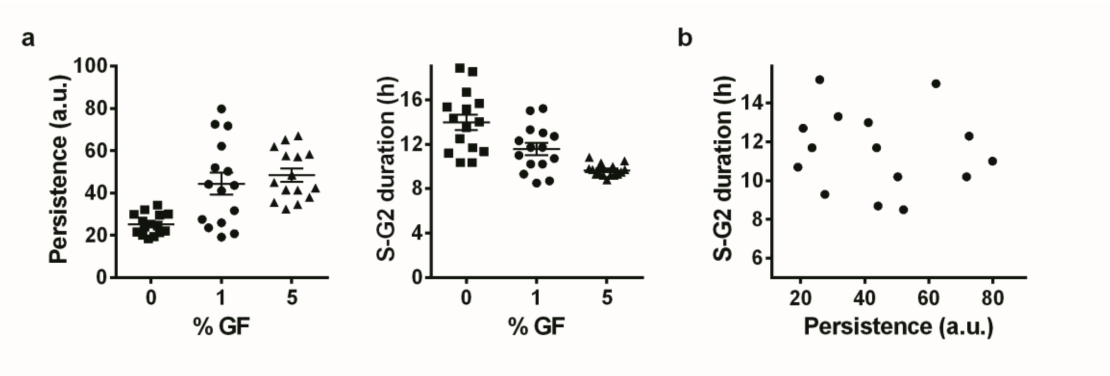

Figure S9: The duration of S and G2 phases is not correlated with migration persistence. a Individual MCF10A-FUCCI cells were specifically tracked during S and G2 phases, from the time of their transition from red to green to the time of cell rounding indicating entry into mitosis. The % of GF affected both the persistence of cell migration and the duration of S and G2 phases, albeit to a smaller extent than the duration of G1 phase (Fig. 3b). As in G1 phase, the distribution of persistence during S and G2 phases is particularly scattered in 1% GF. **c** The duration of S and G2 phases does not scale with migration persistence (*n*=15 cells, 1% GF).
