# Supplementary material for: Cortical branched actin determines cell cycle progression
Source: Cell Res. 2019 Apr 10;29(6):432–45. doi: 10.1038/s41422-019-0160-9 (PMC6796858; doi:10.1038/s41422-019-0160-9)
Supplement: Supplementary file 16 — Supplementary FigureS10 [file 41422_2019_160_MOESM16_ESM.pdf]

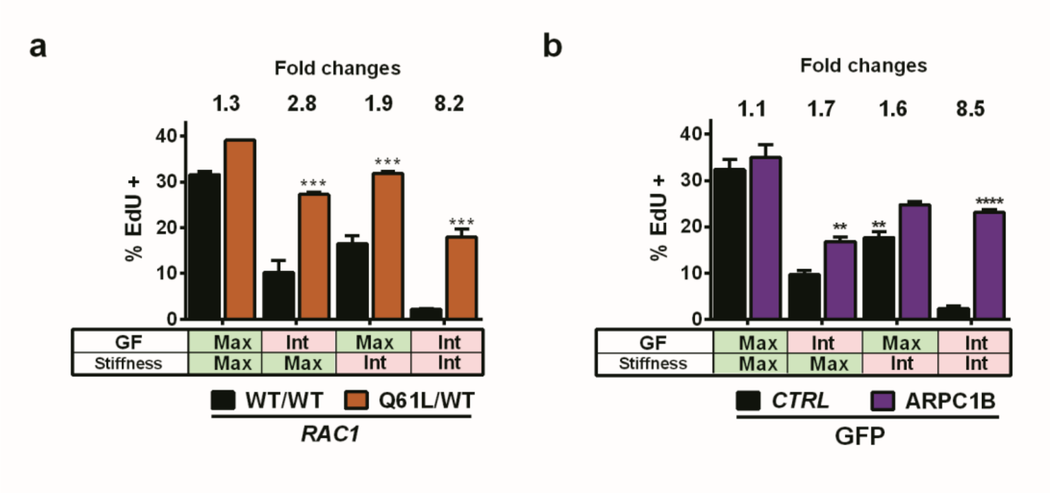

Figure S10: Expression of RAC1 Q61L or overexpression of GFP-ARPC1B greatly enhances cell cycle progression when both growth factor and substratum rigidity are simultaneously suboptimal. These effects are similar to the one of ARPIN depletion displayed in Fig. 5c.
