# Supplementary material for: Cortical branched actin determines cell cycle progression
Source: Cell Res. 2019 Apr 10;29(6):432–45. doi: 10.1038/s41422-019-0160-9 (PMC6796858; doi:10.1038/s41422-019-0160-9)
Supplement: Supplementary file 17 — Supplementary FigureS11 [file 41422_2019_160_MOESM17_ESM.pdf]

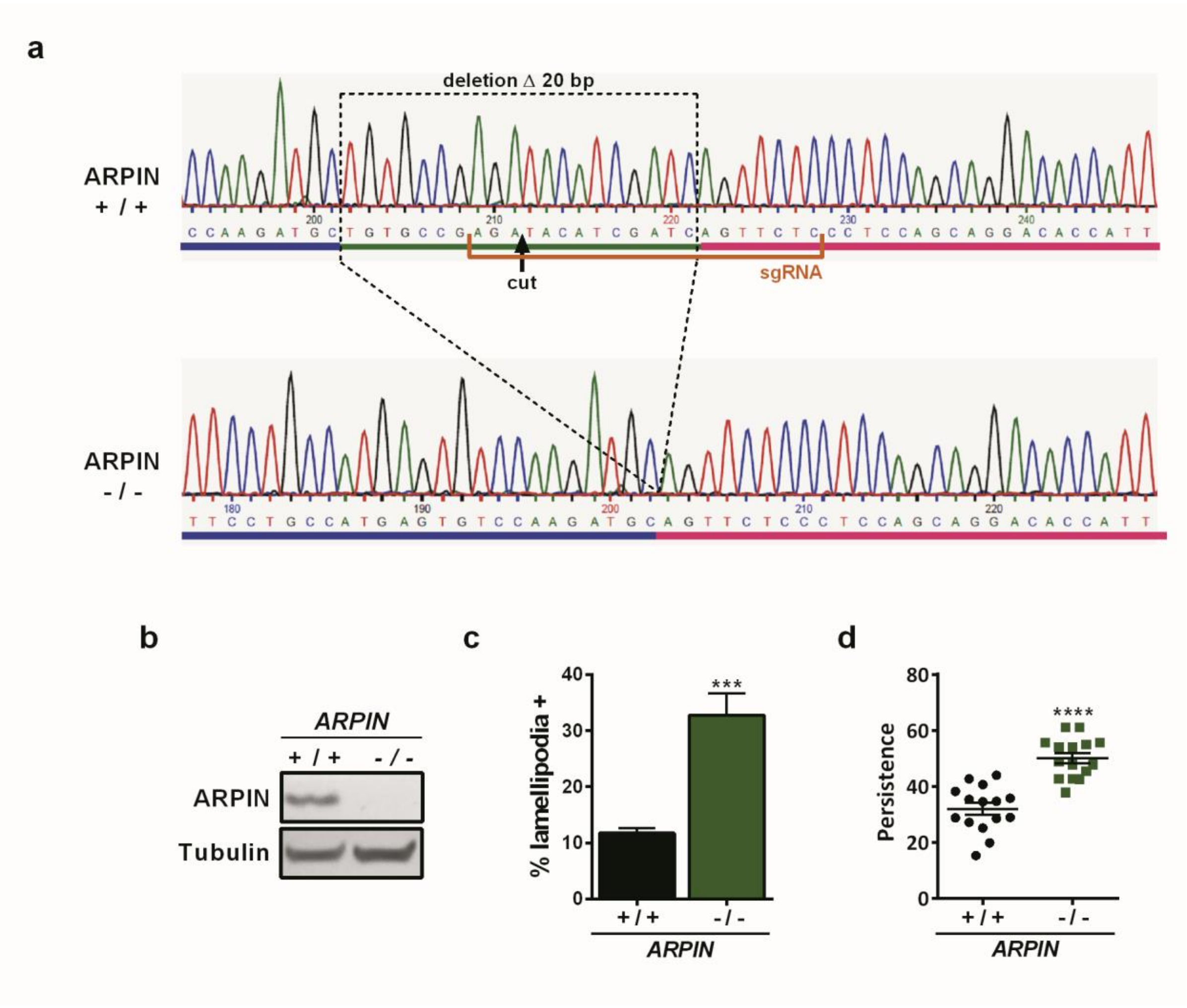

Figure S11: Characterisation of a MCF10A cell line knockout for *ARPIN*. **b** ARPIN knockout cells have been generated with the CRISPR-Cas9 system (*cf.* Methods). The clone used here has a deletion of 20 nucleotides in the two *ARPIN* alleles. **b** The ARPIN protein is not expressed as a result. **c** *ARPIN* knockout cells display more lamellipodia than controls. **d** *ARPIN* knockout cells migrate more persistently than controls.
