# Supplementary material for: Cortical branched actin determines cell cycle progression
Source: Cell Res. 2019 Apr 10;29(6):432–45. doi: 10.1038/s41422-019-0160-9 (PMC6796858; doi:10.1038/s41422-019-0160-9)
Supplement: Supplementary file 18 — Supplementary FigureS12 [file 41422_2019_160_MOESM18_ESM.pdf]

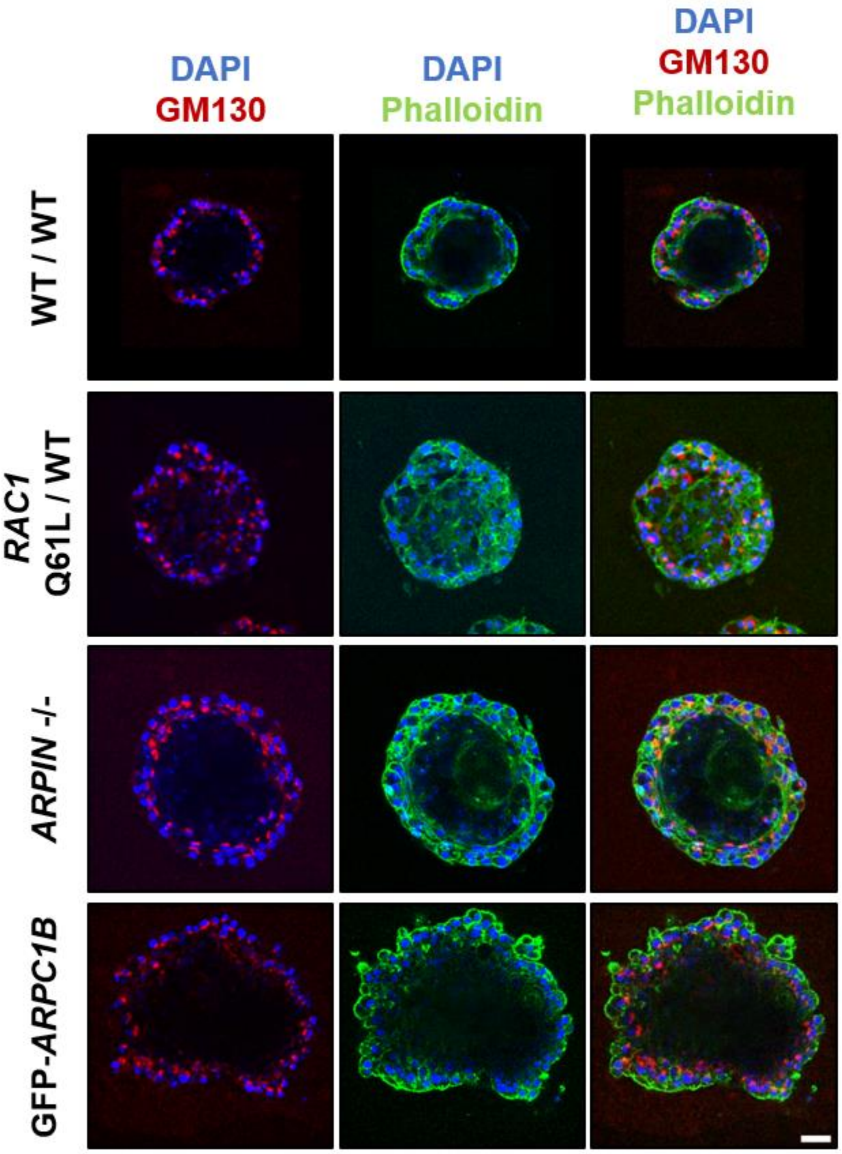

Figure S12: Cell polarity in 3D acini. Acini were stained with DAPI (blue), Phalloidin (green) and with antibodies targeting the Golgi protein, GM130 (red). Phalloidin highlights cell boundaries, whereas GM130 is apically localised, above the nucleus facing the lumen. Confocal microscopy, scale bar : 50 µm.
