# Supplementary material for: Cortical branched actin determines cell cycle progression
Source: Cell Res. 2019 Apr 10;29(6):432–45. doi: 10.1038/s41422-019-0160-9 (PMC6796858; doi:10.1038/s41422-019-0160-9)
Supplement: Supplementary file 19 — Supplementary FigureS13 [file 41422_2019_160_MOESM19_ESM.pdf]

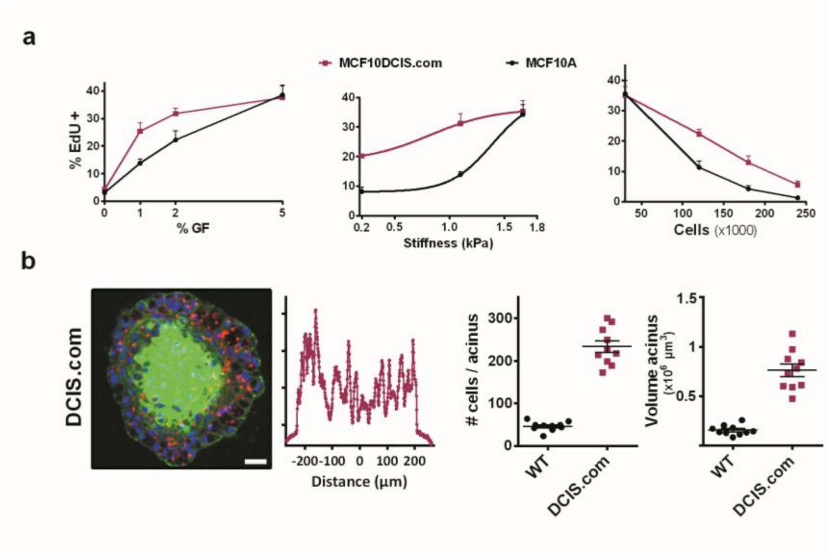

Figure S13: Comparison of transformed MCF10DCIS.com cells with parental immortalised MCF10A cells. a MCF10DCIS.com cells cycle faster than MCF10A cells at intermediate levels of GF, at low substrate stiffness and at intermediate cell densities, as seen in MCF10A cells expressing RAC1 Q61L, GFP-ARPC1B or depleted of ARPIN. **b** MCF10DCIS.com cells also grow more than MCF10A cells in 3D matrigel, but are impaired in the morphogenesis of acini. DAPI in blue, Phalloidin in green and GM130 in red. Confocal microscopy, scale bar : 50 µm.
