# Supplementary material for: Cortical branched actin determines cell cycle progression
Source: Cell Res. 2019 Apr 10;29(6):432–45. doi: 10.1038/s41422-019-0160-9 (PMC6796858; doi:10.1038/s41422-019-0160-9)
Supplement: Supplementary file 20 — Supplementary FigureS14 [file 41422_2019_160_MOESM20_ESM.pdf]

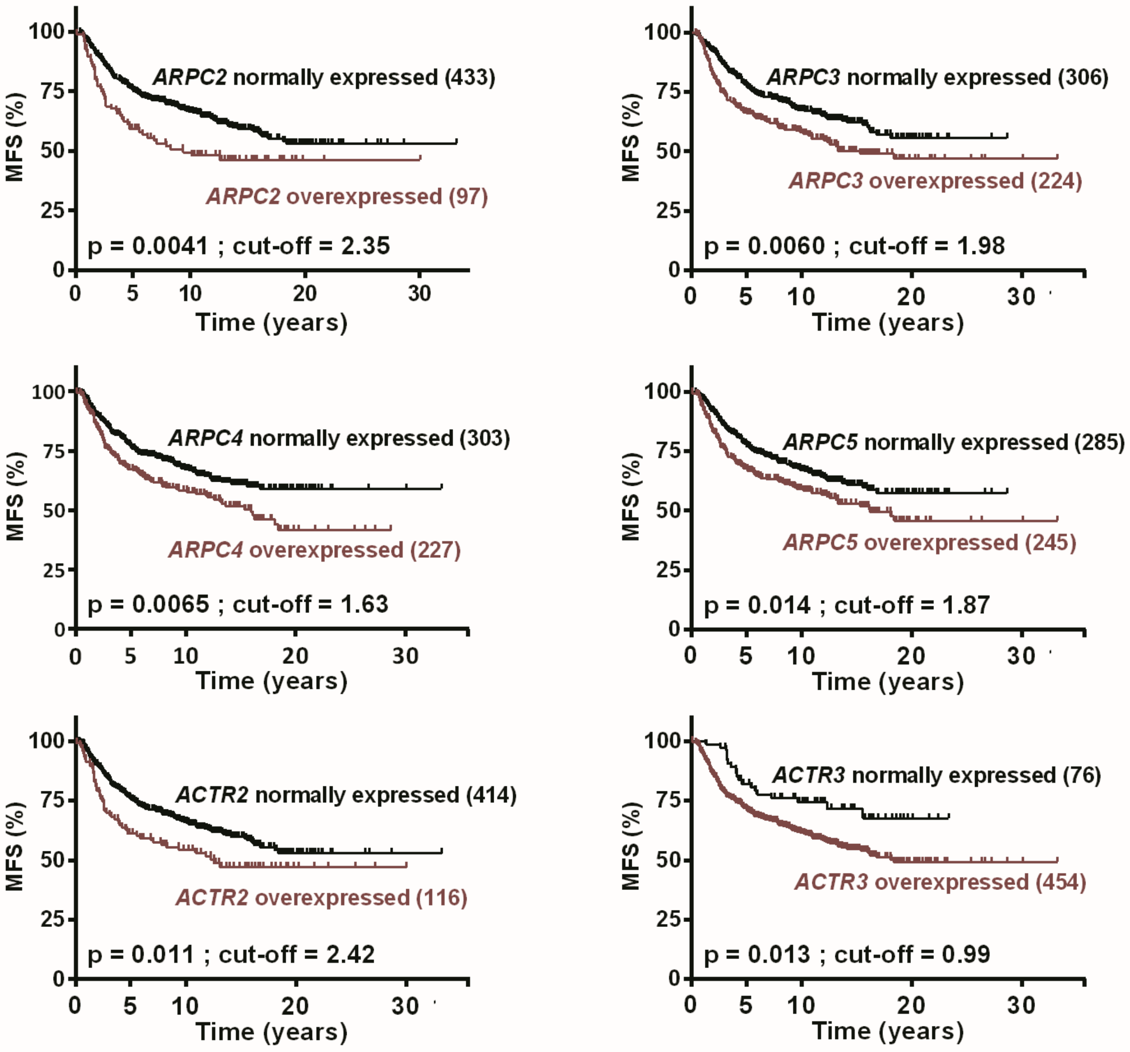

Figure S14: Kaplan-Meier representation of metastasis-free survival (MFS) depending on the overexpression of various Arp2/3 subunits. The univariate p and the optimal cut-off used are displayed on the graphs. None of these Arp2/3 subunits has a prognosis power stronger than the one of ARPC1B.
