# Supplementary material for: Cortical branched actin determines cell cycle progression
Source: Cell Res. 2019 Apr 10;29(6):432–45. doi: 10.1038/s41422-019-0160-9 (PMC6796858; doi:10.1038/s41422-019-0160-9)
Supplement: Supplementary file 21 — Supplementary FigureS15 [file 41422_2019_160_MOESM21_ESM.pdf]

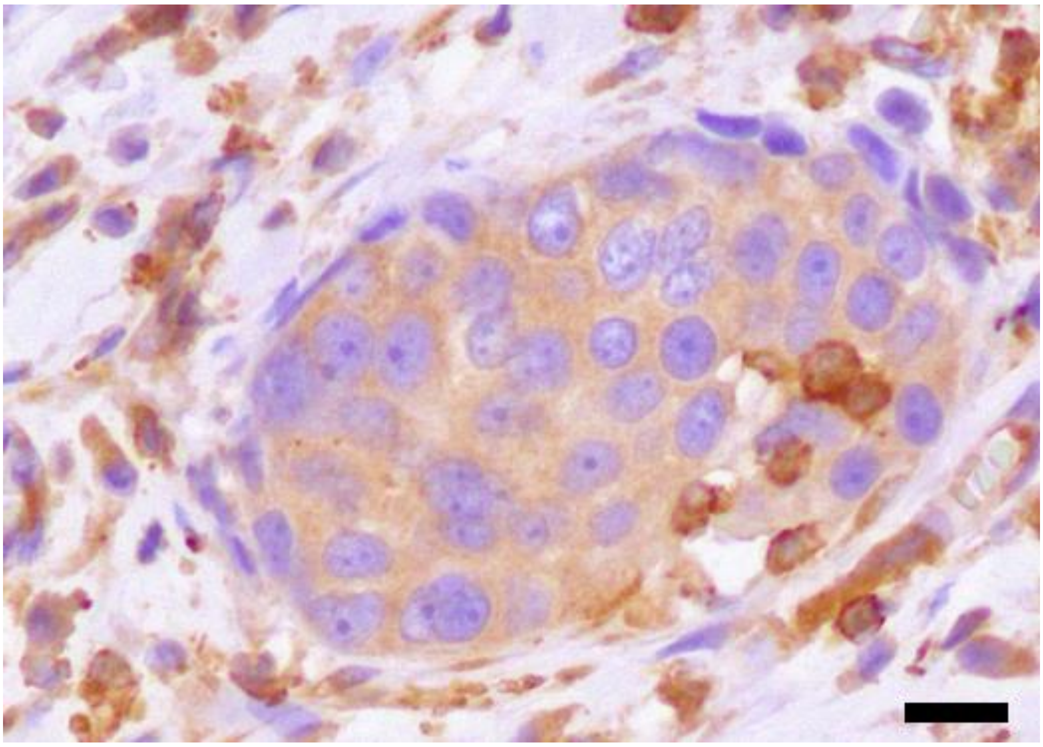

Figure S15: Immunohistochemistry of ARPC1B in mammary carcinoma. ARPC1B (brown) is diffuse in the solid mass of carcinoma cells at the center of the image. The tumour is heterogenous. Some small cells display a bright staining. Scale bar : 20 µm.
