# Supplementary material for: Cortical branched actin determines cell cycle progression
Source: Cell Res. 2019 Apr 10;29(6):432–45. doi: 10.1038/s41422-019-0160-9 (PMC6796858; doi:10.1038/s41422-019-0160-9)
Supplement: Supplementary file 22 — Supplementary TableS1 [file 41422_2019_160_MOESM22_ESM.pdf]

Table S1: Relationships between *ARPC1B* transcript levels and classical clinical biological parameters in a series of 527 breast cancer

	Total population (%)	Number of patients (%)		*p-value* [a]
		ARPC1B mRNA expression ≤5 relative to normals	*ARPC1B* mRNA expression >5 relative to normals	
Total	527 (100)	462 (87.7)	65 (12.3)	
Age				
≤50	125 (23.7)	106 (84.8)	19 (15.2)	0.26 (NS)
>50	402 (76.3)	356 (88.6)	46 (11.4)	
SBR histological grade [b, c]				
I	60 (11.7)	58 (96.7)	2 (3.3)	**0.00011**
II	241 (47.1)	221 (91.7)	20 (8.3)	
III	211 (41.2)	169 (80.1)	42 (19.9)	
Lymph node status [d]				
0	159 (30.5)	139 (87.4)	20 (12.6)	0.52 (NS)
1-3	250 (47.9)	223 (89.2)	27 (10.8)	
>3	113 (21.6)	96 (85.0)	17 (15.0)	
Macroscopic tumour size [e]				
≤25mm	248 (48.0)	223 (89.9)	25 (10.1)	0.20 (NS)
>25mm	269 (52.0)	232 (86.2)	37 (13.8)	
ERα status				
Negative	182 (34.3)	142 (78.0)	40 (22.0)	**0.0000010**
Positive	345 (65.7)	320 (92.8)	25 (7.2)	
PR status				
Negative	256 (48.4)	214 (83.6)	42 (16.4)	**0.0057**
Positive	271 (51.6)	248 (91.5)	23 (8.5)	
ERBB2 status				
Negative	397 (75.3)	361 (90.9)	36 (9.1)	**0.000068**
Positive	130 (24.7)	101 (77.7)	29 (22.3)	
Molecular subtypes				
RH- ERBB2-	102 (19.4)	84 (82.4)	18 (17.6)	**0.0000016**
RH- ERBB2+	73 (13.7)	52 (71.2)	21 (28.8)	
RH+ ERBB2-	295 (56.0)	277 (93.9)	18 (6.1)	
RH+ ERBB2+	57 (11.0)	49 (86.0)	8 (14.0)	
Relapse				
No	317 (60.2)	287 (90.5)	30 (9.5)	**0.014**
Yes	210 (39.8)	175 (83.3)	35 (16.7)	

[a] χ^2Test, [b] Scarff Bloom Richardson classification. Information available for 512 patients (c), 522 patients (d), 517 patients (e).

Supplementary references

1. Gautreau A, Ho H-YH, Li J, Steen H, Gygi SP, Kirschner MW. Purification and architecture of the ubiquitous Wave complex. *Proc Natl Acad Sci U S A* 2004; **101**:4379–4383.

2. Gorelik R, Gautreau A. Quantitative and unbiased analysis of directional persistence in cell migration. *Nature Protocols* 2014; **9**:1931–1943.
